# Plant-derived vesicles: isolation strategies and therapeutic applications

**DOI:** 10.3389/fpls.2025.1660579

**Published:** 2026-01-05

**Authors:** Jiazhong Li, Zhiyi Wang, Feiran Wang, Xiuwei Du, Xiaogang Pang

**Affiliations:** 1 Shandong Technician Institute, Jinan, China; 2 College of Pharmaceutical Science, Shandong University of Traditional Chinese Medicine, Jinan, China; 3 Jinan Third People's Hospital, The Third People’s Hospital of Jinan, Shandong, China; 4 Experimental Center, Shandong University of Traditional Chinese Medicine, Jinan, China

**Keywords:** drug delivery, nanovesicles, plant-derived vesicles, preparation and purification of PDVs, therapeutic applications

## Abstract

Plant-derived vesicles (PDVs) are vesicle-like structures secreted by plant cells, containing components such as membrane proteins、nucleic acids and enzymes. These vesicles play significant roles in plant growth, tissue repair and self-defense. Additionally, PDVs are safe and easy to extract, and they demonstrate promising therapeutic effects on inflammation, cancer and wound healing. Due to their unique properties, PDVs can serve as drug carriers, effectively shuttling through cells to aid disease treatment. In this study, we review the methods for separating and preparing PDVs and highlight their application in disease treatment using nanovesicles derived from plants such as cucumber、broccoli、lemon、turmeric、ginseng、ginger、garlic、grape、yam、tomato and grapefruit. The biomedical applications of PDVs in drug delivery, anticancer therapies, anti-inflammatory responses, antioxidation and wound healing are also introduced. Finally, the feasibility, characteristics, challenges and future prospects of PDVs are discussed.

## 1 Introduction

The International Society for Extracellular Vesicles defines extracellular vesicles (EVs) as ‘particles naturally released by cells without functional nuclei’ (Alzahrani et al., 2023). These vesicles are secreted by all cell types, including those from animals and plants. EVs are membrane-bound structures primarily composed of lipids and contain various biochemically active compounds, such as proteins、lipids、metabolites、sugars、RNA (mRNA, miRNA and siRNA) and even DNA. They facilitate the exchange of materials and information between membranes and can transfer signals from donor cells to recipient cells. Upon recognition by recipient cells, the surface molecules of EVs can activate signaling pathways (Wu et al., 2023). In the 1960s, researchers first identified vesicles derived from carrots (Halperin and Jensen, 1967). Since then, EVs have been reported in various plant species. Plant-derived vesicles (PDVs) can be isolated from fresh vegetable and fruit juices, as well as from Chinese herbal medicines such as ginseng and ginger. PDVs play crucial roles in intercellular communication within plants and in cross-species regulation between plants, animals and microorganisms (Feng et al., 2024). PDVs exhibit diverse biological and pharmacological effects, including wound healing (Nakano and Fujimiya, 2021), antioxidant activity (Li et al., 2021), anti-tumor effects (Sarasati et al., 2023), anti-inflammatory properties (Narauskaite et al., 2021) and potential as drug carriers (Feng et al., 2023). Compared to chemically synthesized nanocarriers, PDVs are safer, non-toxic and capable of crossing the blood-brain barrier (Qiao et al., 2023). As technology advances, increasing evidence suggests that PDVs will become pivotal in the pharmaceutical and biotechnology industries, particularly in treating diseases such as cancer (Wu et al., 2022). Despite their promising applications, several challenges hinder the clinical translation of EVs. These include low yields that limit large-scale clinical use and the potential for PDVs to activate the host’s immune system, which could lead to adverse effects (Hao et al., 2024).

In this study, we review the classification, preparation and identification methods of PDVs. We highlight the application of nanoparticles derived from plants such as ginseng、ginger、garlic、grape、lemon and grapefruit in disease prevention and clinical use. Compared to mammalian cell-derived extracellular vesicles (EVs) or milk EVs, PDVs offer distinct advantages such as higher yield, lower immunogenicity, easier scalability, and the absence of ethical concerns. These attributes position PDVs as a promising alternative for therapeutic and drug delivery applications, warranting focused investigation. Finally, we provide our perspectives on the challenges and opportunities in this field. However, most current studies remain at the descriptive level, focusing on the isolation and phenomenological observations of PDVs efficacy. Critical challenges, such as the lack of standardized characterization protocols, insufficient mechanistic understanding of PDV-cell interactions, limited scalability of production methods, and unclear regulatory pathways for clinical translation, remain largely unaddressed. This review aims not only to summarize the current state of PDVs research but also to critically evaluate these limitations and propose future directions for the field.

## 2 Separation, purification, and characterization of PDVS

### 2.1 Isolation and purification of PDVs

**Figure 1:**
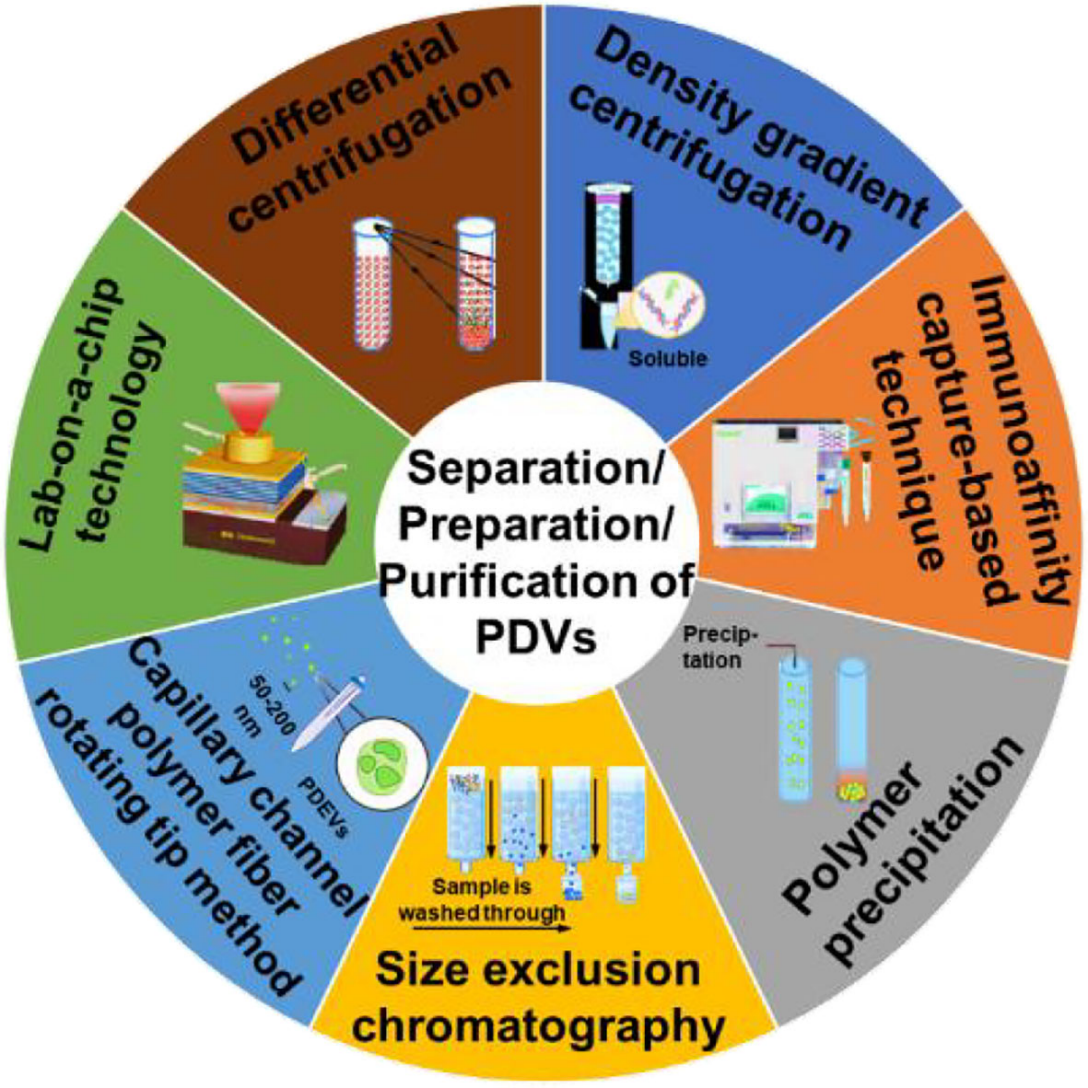
Common separation and purification techniques of exosomes.

The morphology and components of PDVs are heterogeneous, with significant variations in size, density and protein characteristics among different EVs. These differences make it difficult to separate and study PDVs using conventional techniques. Generally, the separation principle relies on the density and size of PDVs, as particles of different sizes exhibit varying sedimentation rates during centrifugation. In practice, the optimal centrifugation speed and time depend on the specific characteristics of the plant species (Yang et al., 2020). The separation and purification of PDVs remain challenging, and no single method is universally suitable. Current methods for preparing PDVs include ultracentrifugation, ultrafiltration, co-precipitation and emerging technologies (**Figure 1**) like size exclusion chromatography (SEC) and immune-affinity enrichment (Kim and Rhee, 2021). This study primarily focuses on the advantages of differential centrifugation, density gradient centrifugation, immunoaffinity capture technology and polymer precipitation methods. Differential centrifugation, known as ‘Gold Standard Technology,’ can process numerous samples in a short time. Density gradient centrifugation efficiently separates substances of different densities in samples. Immunoaffinity capture technology uses surface antigen affinity for effective separation. The polymer precipitation method differs from immunoaffinity capture in that it uses a surface charge to selectively precipitate the target polymer. The following section provides a detailed introduction to these separation methods (**Table 1**).

**Table 1:** Main EV separation methods: advantages and disadvantages .

Method	Principle	Advantages	Disadvantages	Yield	Purity	Scalability	Cost	Common contaminants	References
Differential Centrifugation	Density & size	High throughput, reproducible	Time-consuming, low purity	High	Low	Good	Low	Proteins, cell debris	(Chen et al., 2022; Cong et al., 2022; Wu et al., 2024)
Density Gradient Centrifugation	Density	High purity, resolves subtypes	Laborious, low yield	Medium	High	Poor	Medium	Polysaccharides, starch	(Gurunathan et al., 2021; Barzin et al., 2023; Kathait et al, 2024)
Immunoaffinity Capture	Surface antigen	High specificity, pure subsets	Antibody-dependent, costly	Low	Very High	Poor	High	Non-specific proteins	(Min et al., 2021; Oosterbeek et al., 2021; Yang et al., 2024)
Polymer Precipitation	Solubility	Simple, scalable	Low purity, polymer residue	High	Low	Excellent	Low	Proteins, nucleic acids	(Liu et al., 2021; Liu et al., 2024; Nieszporek et al., 2024)
Size Exclusion Chromatography	Size	High purity, gentle	Sample dilution, time-consuming	Medium	High	Medium	Medium	Proteins, lipoproteins	(Berger et al., 2020; Jia et al., 2022; Shinge et al., 2022)

#### 2.1.1 Differential centrifugation

Differential centrifugation is a prevalent method for separating PDVs, offering high repeatability with minimal impact on samples, which makes it suitable for various types of samples. This method relies on the differing centrifugal forces of components in the centrifugal field to achieve separation, allowing vesicles to be isolated from other substances. It is generally regarded as the ‘Gold Standard Technology’ for separating vesicles or other components (Chen et al., 2022). The process typically involves extracting plant material from roots, stems, fruits and leaves, grinding it into juice, filtering it and then subjecting the collected juice to differential centrifugation. The supernatant is then further processed to isolate vesicles (Zhang et al., 2022; Liu et al., 2024). Zhang et al. demonstrated that sequential low-, medium- and high-speed centrifugation during differential centrifugation effectively isolates highly pure vesicles through ultracentrifugation. This technique enables the segregation and purification of PDVs based on their density and size while efficiently removing large particles, dead cells, mucin, fibers and cellular debris (Wu et al., 2024). The advantage of this method is its ability to process large volumes of samples efficiently, which can achieve “selective sedimentation” by means of rotational speed regulation. It also minimizes damage to vesicles, preserving their integrity and offers the benefits of cost-effectiveness and commercial practicality (Nemidkanama and Chaichanawongsaroj, 2022).

However, differential centrifugation has the drawback of a low recovery rate for foreign bodies and the potential for co-precipitation of protein aggregates, which can affect the purity of the vesicles. In PDV purification, ultracentrifugation is often performed stepwise, from low to high concentration, which can be laborious and inefficient (Cong et al., 2022; Wu et al., 2024). Purity & Quality Control: Purity can be assessed via protein quantification (e.g., BCA assay.

#### 2.1.2 Density gradient centrifugation

Density gradient centrifugation is another commonly used method for separating PDVs. In this technique, a gradient is created using a solution of varying densities, with vesicles of different densities precipitating and separating within the gradient during centrifugation, thus purifying PDVs by removing other vesicles or particles. Typically, a gradient-density separation medium is placed in the centrifugal tube, and the coarse separated sample is loaded on top of the medium. It is then further purified by extended high-speed centrifugation, with the vesicles eventually reaching the isodensity layer and separating from other particles (Barzin et al., 2023). The concentration of particulate matter in distinct nano-pollutants reflects the density of vesicles, which can be separated using differential centrifugal force. To enhance the precision of vesicle isolation, density gradient centrifugation techniques are employed, which not only purify the vesicles but also reduce the likelihood of experimental errors (del Pozo-Acebo et al., 2022). However, the presence of cellulose and starch in plant juice can cause difficulties during centrifugation. To overcome the limitations of this technique, the most commonly used approach is combining differential centrifugation with density gradient centrifugation to achieve high extraction purity (Kathait et al, 2024).

The density gradient centrifugation method has high resolution, allowing the separation of substances with small differences in density, thus enabling efficient separation of materials with different densities in samples (Chen et al., 2022). While density gradient centrifugation is effective for obtaining purer PDVs, it also has drawbacks, including a time-consuming process, limited throughput and the potential for residue from particles with similar densities. The technique is complex and requires a high level of expertise from the experimenters. Purity & Quality Control: Vesicle bands are collected and analyzed via NTA, TEM, and Western blot for specific markers.

#### 2.1.3 Immunoaffinity capture technique

The immunoaffinity capture technique is one method used to isolate and purify certain types of PDVs. The discovery of protein markers on PDVs from different vesicles has enabled the development of specific interactions between these markers and their corresponding antibodies or ligands, providing an efficient separation method for PDVs (Gurunathan et al., 2021). Immuno-affinity capture technology utilizes functional groups on the surface of magnetic beads to specifically recognize PDV surface proteins, significantly enhancing the enrichment of vesicles carrying specific proteins and enabling the separation of PDVs under the influence of a magnetic field. This method allows for the separation of specific PDVs and their subtypes, provided the surface proteins can be specifically recognized and effectively dissociated, with the external magnetic field being easy to adjust and offering a good enrichment effect (Fang et al., 2021).

With the continuous advancement of nanotechnology, immunoaffinity capture technology has seen improvements. For example, Zhang et al. developed a Y-shaped micro-column nanostructure interface to increase the capture area (Wu et al., 2023). Kang et al. designed a device to generate mechanical rotation, increasing the binding probability between antibodies and PDVs (Min et al., 2021). Similarly, immune-affinity capture plays an important role in cancer treatment. Scientists, for example, use antibodies against epithelial cell adhesion molecules as nucleophilic agents to specifically capture cancer-derived subsets of these molecules, thus aiding in cancer treatment (Yang et al., 2024).

The main challenge with this technology is recovering PDVs with minimal structural and functional changes, as non-specific protein binding can compromise the purity of PDVs. Moreover, this method is highly dependent on antibodies, and traditional immunoaffinity binding is often too strong to dissociate under physiological conditions, potentially damaging the structure and activity of foreign bodies and resulting in the loss of valuable PDV information. Hence, it is not widely used in laboratories (Chen et al., 2022). Purity & Quality Control: Specificity is confirmed via ELISA or flow cytometry. Purity is high but yield is low.

#### 2.1.4 Polymer precipitation

Polymer precipitation is a well-established commercial technology for separating and purifying target polymers in solution, offering advantages such as simplicity、speed、low cost and minimal damage to PDVs. PDVs can be isolated from biological fluids by separating small vesicles under standard centrifugation conditions (Oosterbeek et al., 2021). Adjusting the precipitation conditions allows the target polymer to be selectively precipitated while other substances are eliminated, resulting in a relatively pure target. Hydrophilic polymers, such as polyethylene glycol (PEG), disrupt the water layer around nanoparticles, forming a particle-polymer network that promotes co-precipitation with hydrophobic proteins and lipid molecules. Polymer precipitation is easily scalable, independent of specialized equipment and capable of rapidly separating vesicles from various sources (Jia et al., 2022). Kalarikkal et al. employed PEG precipitation to selectively enrich nano-scale EVs from ginger roots, demonstrating that the physical and biological properties of PDVs obtained using this method were similar to those obtained by ultracentrifugation (Kalarikkal et al., 2020).

Although simpler than centrifugation, this method has limitations, including low purity and yield, which restrict its application. Furthermore, while polymer precipitation can produce a large quantity of vesicles, concerns about the purity of the isolate remain. For instance, during the precipitation process, external factors such as changes in pH and temperature can compromise the activity and structure of the target substance, causing the experiment to fail (Nieszporek et al., 2024). Additionally, selective precipitation is challenging in complex mixtures or at low concentrations of the target substance. Some studies have also shown that co-precipitation of pollutants, such as proteins、nucleic acids and other non-vesicular structures, complicates the analysis and prevents the expected experimental outcomes (Liu et al., 2021). Purity & Quality Control: Purity is lower; often requires additional purification steps. Quality is assessed via NTA and protein content.

#### 2.1.5 Size exclusion chromatography

SEC, also known as gel filtration chromatography, is a technique used to separate molecules or particles based on their fluid dynamics volume. It utilizes the selective permeability of a porous fixed-phase gel to achieve efficient separation. Larger molecules or particles cannot enter the separation matrix and are excluded from the system, moving rapidly through it. In contrast, PDVs, which are smaller, can enter the designed solid-phase separation matrix, move a longer distance and experience a time delay, allowing for their separation and extraction (Liu et al., 2024). Since SEC does not require a large sample volume and the shear force generated in the process does not damage the vesicles’ original structure, it preserves the functional and biological activity of EVs. This method also enables the recovery of EVs with high purity and integrity, making it preferable to centrifugation (Sidhom et al., 2020).

In recent years, SEC has become a standard method for separating PDVs due to its high efficiency, simplicity and time-saving qualities. Combining SEC with other techniques offers the highest purity without sacrificing yield. For example, Kang et al. successfully isolated PDVs from cabbage using SEC combined with other methods (Shinge et al., 2022; Kang et al., 2024). Purity & Quality Control: High purity; assessed via NTA, TEM, and Western blot. Common contaminants include proteins and lipoprotein particles.

#### 2.1.6 Other methods

In addition to the abovementioned methods, more scientists are focusing on developing innovative separation technologies for PDVs. Recently, the development of lab-on-a-chip technology has transformed the research landscape in PDV-related fields. Microfluidic-based systems have seen remarkable advancements by integrating various separation methods into microchips, particularly in disease diagnosis (Razaulla et al., 2022; Ren et al., 2024). Another method involves separating and preparing EVs from plants and fruits by rapidly rotating the tips of polymer fibers in capillary tubes. Additionally, *in-situ* imaging technology can enhance imaging resolution, revealing more detailed vesicle transport processes, which benefits experimental operations. These separation methods offer advantages such as small sample volumes、low cost、high throughput and improved accuracy. Furthermore, technologies like asymmetric flow field-flow fractionation and acoustic fractionation are increasingly being used to separate PDVs. However, each method has its strengths and should be selected and optimized based on the specific research objectives and requirements (Jackson et al., 2023).

### 2.2 The characterization of PDVs

As a natural subset of extracellular vesicles, plant-derived vesicles (PDVs) are widely present in plant tissues, bodily fluids, and secretions. Their membrane structures encapsulate a variety of bioactive components such as proteins, nucleic acids, lipids, and small-molecule metabolites, exhibiting enormous application potential in anti-inflammatory, anti-tumor, immunomodulatory, and natural drug delivery fields. The characterization of PDVs is a prerequisite for elucidating their functions and advancing their clinical translation. In this section, we will review PDVs in terms of morphological characterization, physicochemical property characterization, and characterization of functional bioactive components, aiming to provide a reference for the standardized research of PDVs.

#### 2.2.1 The morphological characterization of PDVs

Morphological characterization is the foundation for the identification of PDVs, primarily involving the observation of vesicle morphology, size distribution, and integrity. Currently, the main techniques for morphological characterization include transmission electron microscopy (TEM), scanning electron microscopy (SEM), atomic force microscopy (AFM), and cryogenic transmission electron microscopy (cryo-TEM) (Li et al., 2025).

TEM is the gold standard for PDVs morphological observation, which requires negative staining techniques to clearly reveal the spherical or quasi-spherical structure, bilayer membrane characteristics, and internal hollow morphology of vesicles (Muhammad et al., 2025). SEM is mainly used to observe the surface morphology and aggregation state of PDVs, and is often used complementarily with TEM to comprehensively investigate the structural characteristics of vesicles. Cryo-TEM preserves the intact morphological structure in a three-dimensional imaging mode by rapidly freezing samples, enabling a more realistic reflection of the detailed membrane structure of PDVs; however, it suffers from high equipment costs and complex sample preparation. In addition to providing the morphological structure of PDVs, AFM can also obtain characteristics such as their adhesion, elasticity, and deformability (Priglinger et al., 2021). To date, TEM remains the most commonly used technique for PDV characterization.

#### 2.2.2 The physicochemical property characterization of PDVs

The characterization of the physicochemical properties of PDVs mainly includes size distribution, zeta potential, and polydispersity index (PDI), which are key indicators for evaluating the stability of preparation processes and batch-to-batch consistency of PDVs (Yang et al., 2025) Dynamic light scattering (DLS) analysis can be used to characterize the size distribution and polydispersity index of PDVs. Zeta potential reflects the colloidal stability of PDVs on the one hand, and on the other hand, the rate of their cellular membrane uptake can be evaluated by determining the positive or negative nature of the potential.

#### 2.2.3 The characterization of functional bioactive components of PDVs

As the molecular basis of PDV biological functions, functional bioactive components are characterized using electrophoresis and mass spectrometry techniques (Langellotto et al., 2025). For protein profiling, SDS-PAGE coupled with LC-MS/MS enables comprehensive identification of PDV protein composition; RNA-seq and qRT-PCR allow accurate quantification of RNA components in PDVs; and GC-MS/LC-MS/MS facilitate the systematic characterization of lipid species (e.g., phospholipids, triglycerides, sterols) and small-molecule metabolites (e.g., terpenes, flavonoids, phenolic acids), which are critical for elucidating PDV functional mechanisms.

## 3 Some common PDVs

In PDV research, the focus has shifted towards studying their composition and function, gradually revealing their role in plant growth, development, stress response and signal transduction. As the understanding of PDV composition and functionality matures, research into the medicinal value of PDVs for medical treatments has expanded. PDVs are emerging as a new therapeutic strategy, such as nanocarriers, offering alternative approaches for treating inflammation, cancer and other diseases. The following section will introduce some common PDVs (**Table 2**).

**Table 2:** Common PDVs.

Plant source	Type of PDV	Key applications	Mechanism of action	References
Ginseng	GDNs	Osteoporosis, melanoma, Alzheimer’s disease	Inhibits osteoclasts, promotes apoptosis, neuronal differentiation	(Cao et al., 2019; Yu et al., 2020; Seo et al., 2023; Wang et al., 2024)
Ginger	GDEVs	Lung injury, alcoholic liver injury, inflammatory bowel disease (IBD)	Inhibits viral replication, ROS reduction, anti-inflammatory	(Man et al., 2021; Teng et al., 2021; Zhao et al., 2023; Bahri et al., 2024; Zeng et al., 2024)
Garlic	GDVs	Obesity, non-alcoholic fatty liver disease, (NAFLD) chronic inflammation	Inhibits NLRP3, regulates PFKFB3	(Liu et al., 2021; Sundaram et al., 2022; Liu et al., 2023)
Grape	GENs	Colitis	Promotes intestinal stem cells, Wnt/β-catenin pathway	(Ju et al., 2013)
Grapefruit	GNVs	Vascular calcification, wound healing, melanoma	M2 polarization, ROS reduction, cell cycle arrest	(Wang et al., 2014; Zhuang et al., 2016; Stanly et al., 2020; Savci et al., 2021; De Koning et al., 2022; Feng et al., 2023)
Cucumber	CDNVs	Lung cancer, hypertrophic scars	Enhances cucurbitacin B bioavailability, PDT	(Chen et al., 2022; Kong et al., 2024)
Lemon	LDEVs	Ovarian cancer, kidney stones, glioma	Overcomes MDR, inhibits ER stress, immunomodulation	(Xiao et al., 2022; Zhang et al., 2023; Li et al., 2024)
Broccoli	BDEVs	Constipation, colorectal cancer	miRNA delivery, reverses 5-FU resistance	(del Pozo-Acebo et al., 2022; Tianchi et al., 2023; Cao et al., 2024)
Turmeric	TNVs	Diabetic wounds, colitis	Antioxidant, anti-inflammatory, epithelial repair	(Gao et al., 2022; Wu et al., 2024)
Tomato	Tomato EVs	Intestinal dysbiosis, wound healing	Promotes probiotics, enhances cell migration	(Lee et al., 2023; Daniello et al., 2024)
Yam	YNVs	Osteoporosis	Promotes osteoblast differentiation	(Hwang et al., 2023)
Aloe	ADENHs	Oxidative stress diseases	Activates Nrf2/HO-1 pathway	(Pan et al., 2024)

### 3.1 Ginseng

**Figure 2:**
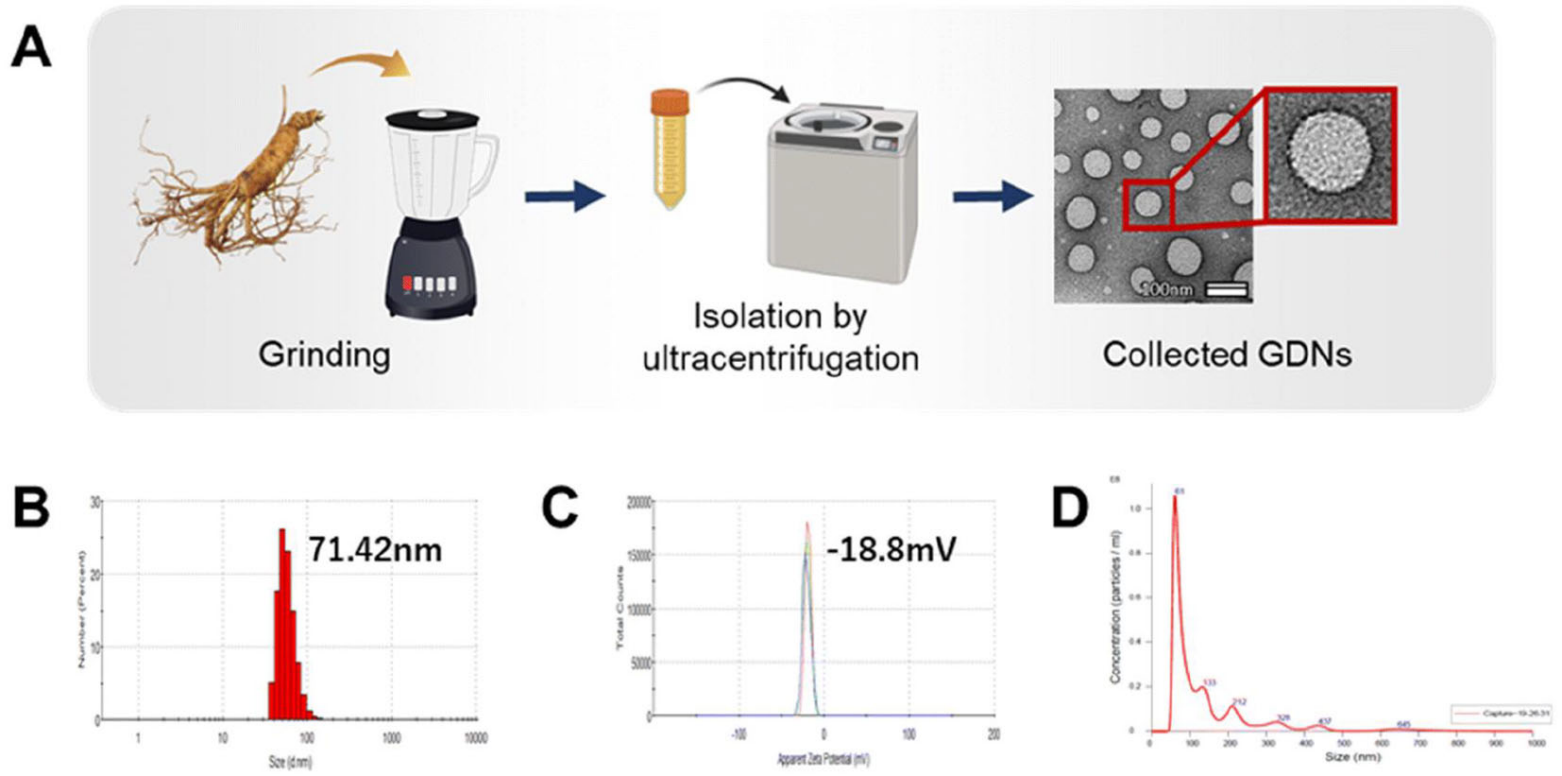
Schematic diagram of the preparation and characterization of ginseng-derived nanovesicles (GDNs). **(A)**. Preparation of GDNs. **(B, C)**. Size distribution and zeta potential of GDNs determined by DLS and **(D)**. NTA analysis. Reprinted with permission from Ref (Seo et al., 2023). Copyright (2023) Royal Society of Chemistry.

Ginseng, known as the ‘King of Herbs,’ is a traditional remedy with a sweet yet slightly bitter taste, commonly used to treat deficiencies in qi, blood, yin and body fluids. The therapeutic effects of ginseng vary depending on its region of origin. PDVs have recently emerged as an effective tool for promoting wound healing, thus aiding tissue repair and regeneration. Researchers have found that ginseng-derived nanovesicles (GDNs) can support brain nerve differentiation and wound healing, offering new insights for treating nerve diseases and promoting tissue regeneration. Osteoporosis, a common condition among older adults that significantly impacts their health, can also benefit from GDNs, which promote bone cell differentiation. To address this issue, Kwansung et al. developed a mouse model to isolate and prepare ginseng vesicles (**Figure 2**), demonstrating that GDNs inhibit osteoclast differentiation, suggesting their potential in treating osteoporosis (Seo et al., 2023).

To better observe the structure of GDNs, Cao et al. purified ginseng by first removing large particles and fibers through centrifugation, followed by ultracentrifugation of the supernatant. The precipitated components were analyzed over time. Transmission electron microscopy revealed the spherical structure of GDNs, providing a novel approach for developing GDNs as drug-delivery carriers. GDNs were then injected into mice, with three groups tested in parallel. The results showed that GDNs significantly promoted the differentiation of mouse melanoma tumor cells, leading to apoptosis and enhancing the anti-tumor response. This discovery may pave the way for a new class of nanodrugs in tumor immunotherapy. The anticancer mechanism of GDNs involves the regulation of macrophage polarization toward the M2 phenotype, which alters the tumor microenvironment and inhibits melanoma growth (Cao et al., 2019).

Personalized cancer vaccines for tumor patients offer a promising approach to addressing tumor heterogeneity and preventing recurrence or metastasis. However, triggering immune activation is challenging due to the weak immunogenicity of autologous tumor antigens. To overcome this, a delivery system for immune adjuvants is needed to enhance the adaptive immune response. Wang et al. developed functional hybrid vesicles (HM NPs) by combining ginseng-derived extracellular vesicle-like particles with membranes from excised autologous tumors. These HM NPs can strengthen specific immune responses to suppress tumor recurrence and metastasis (Wang et al., 2024). Additionally, GDNs have been shown to promote the neuronal differentiation of mesenchymal stem cells, improving dysfunction in mice with Alzheimer’s disease (Yu et al., 2020).

### 3.2 Ginger

Ginger, a medicinal and edible plant, has traditionally been used to treat acupoints in folk medicine. Over the past few decades, it has been employed to treat various ailments, including colds and inflammation. With technological advancements, researchers have discovered that ginger vesicles also play an important role in medicine. The therapeutic potential of ginger-derived exosomal vesicles (GDEVs) lies in their ability to carry intrinsic compounds, which have significant clinical potential. GDEV is a spherical vesicle secreted by the root of ginger, rich in active ingredients (AIs) such as gingerol and ginger oil, which offer numerous health and medical benefits. Due to the hard texture of ginger, most researchers first crush it using a wall-breaking machine or blender, then filter it and extract the vesicles through methods like centrifugation. Differential centrifugation is one of the fundamental techniques for extracting ginger vesicles (Kalarikkal et al., 2020).

During the COVID-19 pandemic, ginger played an indispensable role. Teng et al. demonstrated that GDEV could inhibit lung inflammation caused by EVs released by SARS-CoV-2. Notably, the miRNA in GDEV does not share the same sequence as miRNA derived from virus host cells, inhibiting the expression of viral genes and virus replication. The miRNA cargo in GDEVs plays a crucial role in inhibiting SARS-CoV-2 replication by targeting viral genes, demonstrating cross-kingdom regulation mechanisms (Teng et al., 2021). Man et al. extracted GDEV using ultra-high-speed centrifugation and found that its loading capacity significantly enhanced the liposolubility of gingerol compounds, promoting their intestinal absorption and transport. Experimental research confirmed that GDEV has a good loading capacity and holds significant promise as a carrier of drug-delivery systems (Man et al., 2021).

Zhang et al. demonstrated that GDEV has a protective effect against alcohol-induced liver injury through experimental research. Fresh ginger was collected before the experiment, washed and ground into juice. The ginger juice was then centrifuged in sequence, and after separating the supernatant from the sediment, the protein content was determined using a BCA assay kit. Zhang et al. subsequently conducted experiments on a mouse model of alcoholic liver disease. After euthanising the mouse, serum and liver samples were collected for analysis. The results showed that GDEV-mediated activation of nuclear factor erythroid 2-related factor 2 induced the expression of liver detoxification genes and inhibited the production of reactive oxygen species (ROS), providing some protection to the liver.[GDEVs exert their anti-inflammatory effects through the nuclear factor erythroid 2-related factor 2 (Nrf2) pathway, which induces the expression of liver detoxification genes and inhibits ROS production (Zhao et al., 2023). Inflammatory bowel disease (IBD) is a gastrointestinal condition characterized by chronic diarrhea, which can lead to stomach spasms in severe cases. Studies have shown that oral administration of GDEV can improve the intestinal mucosa and alleviate acute inflammation in an IBD mouse model. Data indicate that GDEV reduces the expression of the inflammatory factor TNF-α, inhibiting tumor growth and chronic colitis while promoting intestinal healing (Bahri et al., 2024). Teng et al. also concluded that GDEV-coated drugs are preferentially absorbed by intestinal bacteria when entering the intestine, thereby shaping the intestinal environment, enhancing the intestinal barrier function to alleviate colitis and offering a novel approach for the clinical treatment of diseases (Zeng et al., 2024).

### 3.3 Garlic

Garlic is a common flavoring agent in daily life, valued not only for its edibility but also for its medicinal properties, such as anti-tumor、anti-hyperlipidaemic and anti-infection effects. Garlic-derived exosome-like nanoparticles (GDVs) are empty vesicles formed by small molecular compounds secreted by garlic cells and have been widely used in the prevention and treatment of various diseases for preventing and treating different diseases (Bian et al., 2023).

Obesity is becoming a global epidemic, and understanding its mechanisms remains a challenge. Sundaram et al. injected GDVs into mice and found that GDVs inhibited systemic and cerebral inflammation, reversing high-fat diet-induced obesity. The researchers showed that GDVs preferentially entered microglia and inhibited brain inflammation in high-fat diet mice, reducing obesity induced by unhealthy diets (Sundaram et al., 2022). The nucleotide-binding domain and leucine-rich repeat family pyrin domain containing 3 (NLRP3) inflammasome plays a key role in the development of several complex inflammatory diseases, including obesity, Alzheimer’s disease and atherosclerosis. Liu et al. discovered that GDVs exhibited effective anti-NLRP3 inflammasome activity in cell cultures and evaluated this function in mouse models of acute liver injury and diet-induced obesity. In these models, GDVs alleviated local inflammation in mice. When administered orally or intravenously, GDVs accumulated in specific tissues, inhibiting NLRP3 inflammasome activation and chronic inflammation induced by diet. As an effective inflammasome inhibitor, GDVs offer a promising new candidate for treating inflammation. GDVs preferentially enter microglia and inhibit brain inflammation through modulation of the NLRP3 inflammasome, which plays a key role in obesity-related neuroinflammation (Liu et al., 2021; Sundaram et al., 2022). The specific targeting of GDVs to inflammatory tissues suggests receptor-mediated uptake mechanisms that warrant further investigation.

GDVs can also be used to treat non-alcoholic fatty liver disease (NAFLD), with their mechanism of action involving the inhibition of inflammatory reactions by regulating the expression of 6-phosphofructo-2-kinase/fructose-2,6-bisphosphatase-3 (PFKFB3). This, in turn, alleviates liver cell dysfunction in mice fed a high-fat diet through macrophage–liver cell regulation. Liu et al. established a NAFLD mouse model, fed the mice GDV-treated feed and measured the expression of PFKFB3 in their livers. The results showed a significant decrease in PFKFB3 expression, indicating that GDVs could improve the symptoms of NAFLD. This study also offers new insights into the molecular mechanisms underlying interspecific communication of exosomes from edible plants (Liu et al., 2023).

### 3.4 Grape

Grapes are a common fruit that are widely available in daily life. They have been valued for thousands of years due to their high medicinal value, including their ability to replenish qi and blood, strengthen bones and muscles and combat fatigue. Grapes contain many vesicle structures composed of lipids, and the PDVs found in grapes have diverse applications in the medical field. Grape-derived exosome-like nanoparticles (GENs) are small membrane vesicles released by grape cells, rich in active components such as phenols and flavonoids, which offer various health benefits and pharmacological effects. GENs can be used for targeted drug delivery, improving utilization and enhancing therapeutic efficacy (Anusree et al., 2024). Teng et al. studied the physiological function of GENs by first grinding grapes and extracting the juice using a blender, followed by filtration through a sieve or gauze. Large grape fibers were removed by differential centrifugation, and the supernatant was concentrated by ultracentrifugation. The GENs were then purified, with the concentrated solution subjected to ultracentrifugation in a discontinuous sucrose gradient for 2 h to remove proteins, other vesicles and RNA aggregates, resulting in a higher purity of GENs (Teng et al., 2022). Studies have shown that intestinal stem cells are the only stem cells capable of producing long-lived intestinal organ structures *in vitro*. Under physiological conditions, GENs can target intestinal hepatocytes *in vivo* through intragastric administration in mice. By promoting the proliferation of intestinal Lgr5 stem cells and activating the Wnt-β-catenin pathway, GENs can induce the proliferation of intestinal stem cells, restore intestinal length and villus height, accelerate mucosal epithelial regeneration and improve intestinal structure. This process helps protect mice from colitis induced by sodium dextran sulfate and aids in the restoration of the entire intestinal structure (Ju et al., 2013).

### 3.5 Grapefruit

Grapefruit (Citrus paradisi), an edible citrus fruit, contains a wealth of organic acids, amino acids and abundant flavonoids with antioxidant and anti-inflammatory properties (Devi et al., 2024). PDVs are widely used in disease treatment due to their excellent biocompatibility and biodegradability (Kim et al., 2022; Lian et al., 2022). Grapefruit-derived nanovesicles (GNVs) exhibit distinct wound-healing, anti-inflammatory and anticancer activities (Wang et al., 2018; Yang et al., 2024).

**Figure 3:**
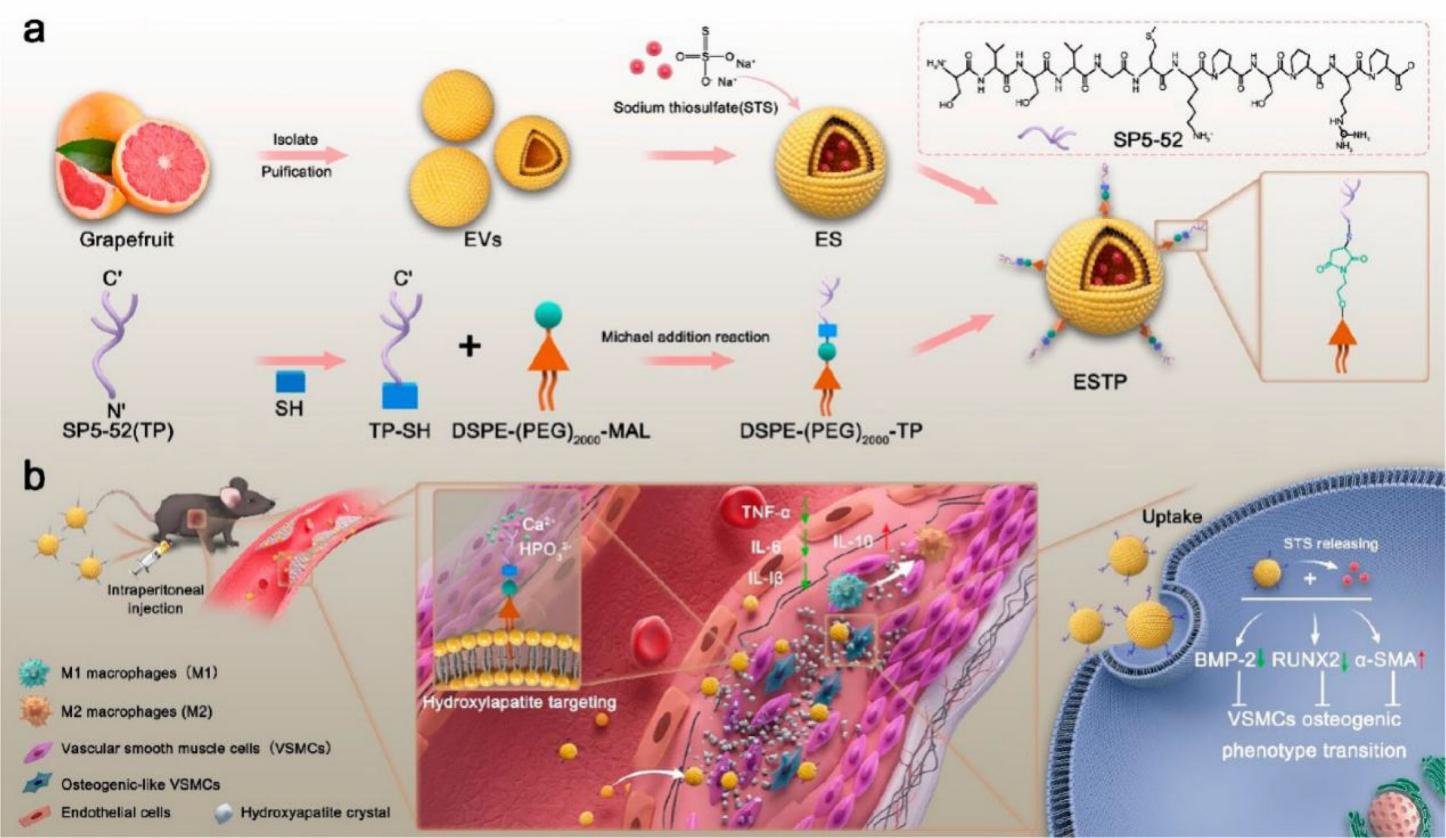
Schematic illustration of ESTP for suppressing vascular calcification (VC). **(a)** Construction of grapefruit-derived extracellular vesicle (EV) nanodrugs (marked as ESTP). **(b)** Mechanism of ESTP against VC. Reprinted with permission from Ref (Feng et al., 2023). Copyright (2024) American Chemical Society.

Sodium thiosulfate (STS), a clinically approved drug for vascular calcification (VC), is hindered by poor bioavailability and significant adverse effects (De Koning et al., 2022). Feng et al. constructed grapefruit-derived extracellular vesicles (ESTP), modified with a hydroxyapatite crystal–binding peptide, for VC-targeted delivery of STS (**Figure 3**). *In vitro*, ESTP demonstrated excellent cellular uptake by calcified vascular smooth muscle cells (VSMCs) and inhibited VSMC calcification. In a VC mouse model, ESTP showed the highest accumulation in calcified arteries compared with other treatment groups. Mechanistically, ESTP prevents VC by driving M2 macrophage polarization, reducing inflammation and inhibiting the bone–vascular axis. This study suggests that biomimetic grapefruit-derived EVs will become a promising drug for VC (Feng et al., 2023).

Chronic wounds are widespread, and wound care remains a major burden on the global healthcare system. Developing safe and effective wound care agents is an urgent issue for biomedical researchers. Plants have a long history of use in wound treatment, and plant-derived EVs are widely utilized in therapeutic research due to their biocompatibility and biodegradability. Grapefruit contains bioactive compounds with anti-inflammatory and antioxidant properties, making it useful for improving wound healing. Savci et al. prepared GNVs as a promising cell-free therapeutic tool for wound healing. In their study, GNVs significantly increased cell viability and migration while reducing intracellular ROS production. Additionally, GNV treatment enhanced the tube-forming ability of treated HUVEC cells. These studies suggest that GNVs can be used as wound-healing agents (Savci et al., 2021).

In addition to their anti-inflammatory, antibacterial and wound-healing properties, GNVs also show great potential in cancer treatment. In one study, GNVs caused cell cycle arrest at the G2/M checkpoint, which was linked to a decrease in the expression levels of cyclins B1 and B2 and an upregulation of the cell cycle inhibitor p21. GNVs also exhibited distinct metabolome profiles and anticancer activities in the A375 human melanoma cell line (Stanly et al., 2020). Effectively delivering drugs to the brain remains a major challenge in central nervous system drug development. Zhuang et al. developed GNVs to carry miR17 for the treatment of mouse brain tumors. The study showed that GNVs modified with folic acid (FA-GNVs) enhanced targeting of the GNVs to folate receptor-overexpressed GL-26 brain tumors. miR17 carried by FA-GNVs was rapidly delivered to the brain through intranasal administration and selectively taken up by GL-26 tumor cells (Zhuang et al., 2016).

Additionally, GNVs have been found to serve as immunomodulators in the intestine, helping to maintain intestinal macrophage homeostasis and reduce the incidence of human diseases. Studies have shown that GNVs are selectively absorbed by intestinal macrophages, improving colitis in mice. The intestinal macrophages selectively ingest GNVs, upregulate the expression of heme oxygenase-1 and inhibit the production of pro-inflammatory cytokines, thereby alleviating colitis. These findings demonstrate that GNVs can function as immune modulators in the intestine, maintaining intestinal macrophage homeostasis (Wang et al., 2014).

Wang et al. prepared grapefruit-derived nano-vectors with a relatively uniform size from grapefruit nanoparticles using the well-known liquid-liquid extraction method. The nanoparticles were then treated with ultrasound, and the lipid was reassembled into a multilayer, flower-like structure around 200 nm in size through a high-pressure homogeniser. GNVs can be used to enhance drug-delivery efficiency and can also be modified to achieve specific cellular targeting (Wang et al., 2013).

### 3.6 Cucumber

Cucumber is a common vegetable in the Cucurbitaceae family, rich in vitamin C, minerals and dietary fiber, with antioxidative, digestive-promoting and moisturizing properties. It is widely used in various dishes and is an important part of the daily diet. Cucurbitacin B (CUB) in cucumber is reported to inhibit the development of lung cancer, liver cancer and albinism. However, due to its low solubility and bioavailability, CUB is not widely used to treat cancer and other diseases. PDVs have been considered effective nanocarriers to improve drug delivery, and cucumber-derived vesicles (CDVs) are attracting increasing attention.

Abraham et al. isolated CDVs using the classical EVs isolation method and high-pressure homogenization to evaluate the dermal penetration efficacy of a lipophilic AI. The study showed that CDVs enhanced the dermal penetration efficacy of the AI surrogate by approximately 200% (Abraham et al., 2022).

**Figure 4:**
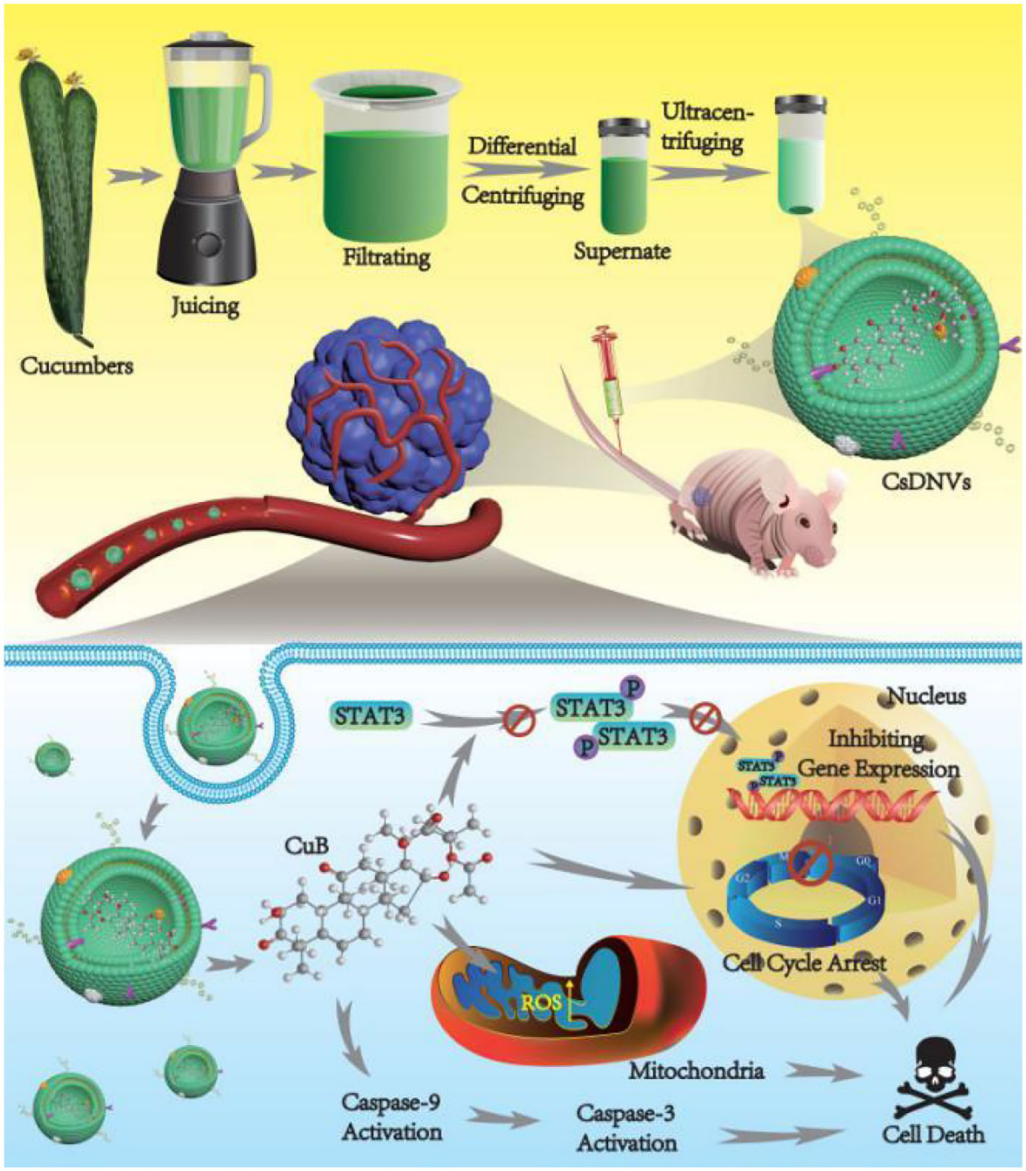
CsDNVs obtained by juicing and centrifugation were used to inhibit tumor development in nude mice. Reprinted with permission from Ref (Chen et al., 2022). Copyright (2022) Dovepress.

Researchers have hypothesized that cucumber components contain CUB, leading to the suggestion that cucumber-derived nanocapsules (CDNVs) may naturally carry CUB. Chen et al. first separated CDNVs through simple juicing and centrifugation, finding that they were less toxic and safer than free CUB (**Figure 4**). The anticancer mechanism was further explored, revealing that CDNVs could inhibit the signal transducer and enhance ROS production, thus improving the bioavailability of CUB. In a mouse model, one group received CUB loaded in CDNVs, while the other group was injected with free CUB to observe cancer cell numbers. The results showed that CUB loaded in CDNVs was safer and more effective in inhibiting cancer than free CUB (Chen et al., 2022).

Hypertrophic scar (HS) is a skin condition characterized by persistent dermal fibrosis due to excessive proliferation and apoptosis of fibroblasts. It often causes varying degrees of itching, as well as appearance and psychological issues, significantly affecting patients’ quality of life. While laser therapy and cryotherapy are common treatments, they come with the risk of new scar tissue formation, surgical complications and vascular injury. Photodynamic therapy (PDT) has recently emerged as a promising treatment with fewer side effects. PDT uses photosensitisers to produce ROS under appropriate light irradiation, which induces apoptosis in hyperproliferative fibroblasts and remodels collagen fibers. A multifunctional biomimetic nano-platform (NDs@EV-RGD), formed by RGD-modified CDVs and copper-based organic metal (Cu-MOF-NDs) composites, has been developed for PDT-mediated HS treatment. NDs@EV-RGD demonstrates remarkable skin permeability, offering a novel strategy for transdermal delivery of nanocarriers. Studies show that when irradiated with near-infrared laser, NDs@EV-RGD generates abundant ROS, significantly enhancing PDT and inducing apoptosis in hyperproliferative fibroblasts, improving transdermal drug-delivery efficiency (Kong et al., 2024).

### 3.7 Lemon

**Figure 5:**
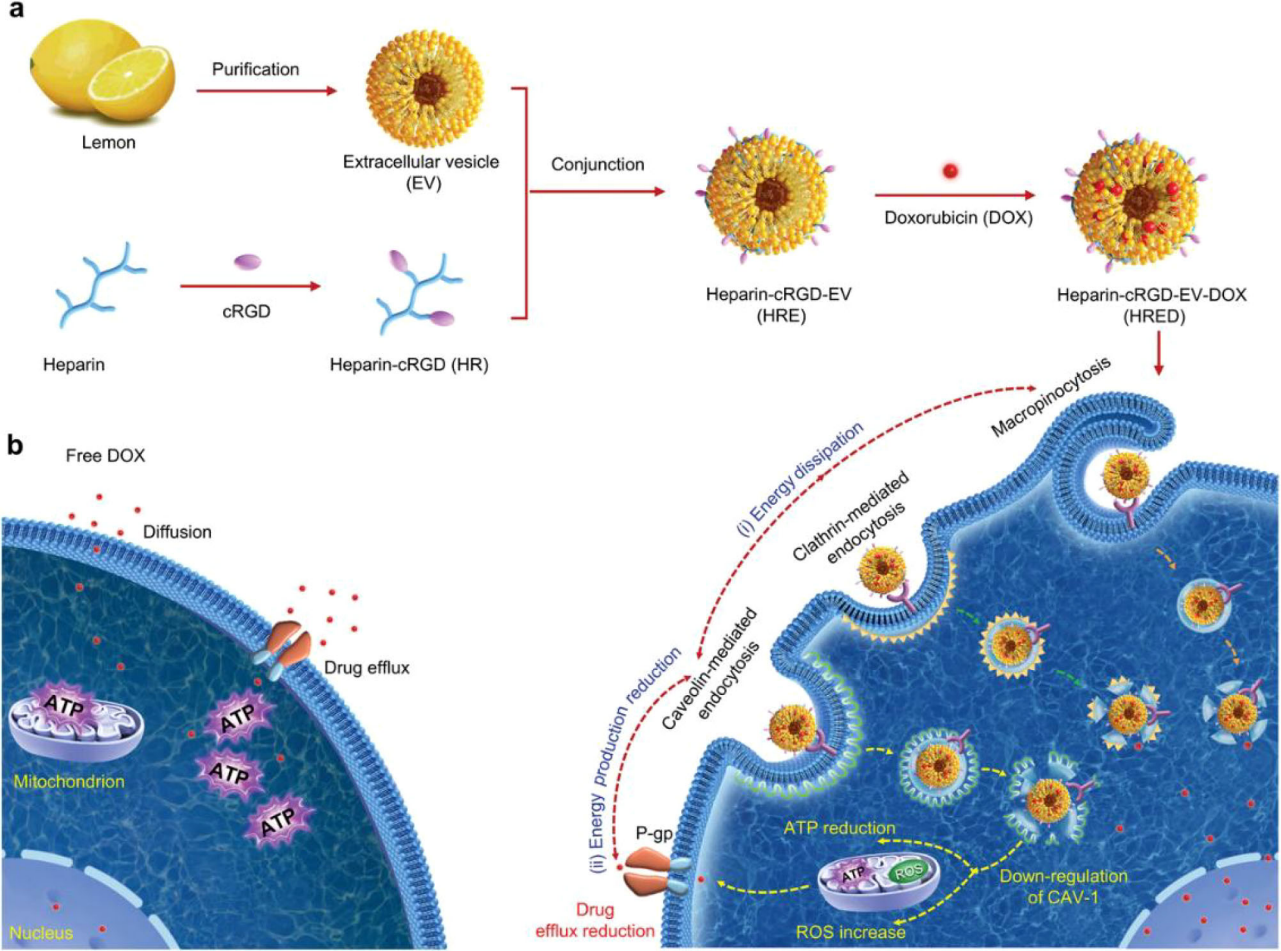
Schematic illustration of lemon-derived extracellular vesicle (LDEV) nanodrugs for overcoming cancer multidrug resistance. **(A)** Preparation of LDEV nanodrugs (marked as HRED). **(B)** HRED Overcome Cancer Multidrug Resistance. Reprinted with permission from Ref (Xiao et al., 2022). Copyright (2022) Wiley.

Lemon is a common citrus fruit rich in anti-inflammatory, antioxidant and anticancer AIs, including vitamin C, vitamin E and hesperidin. Lemon-derived EVs (LDEVs) are stable in the gastrointestinal tract due to the similarity in pH between lemons and the stomach and have been used in the treatment of various diseases (Yang et al., 2020, 82, Tinnirello et al., 2024, Urzi et al., 2023). Xiao et al. Heparin constructed a biomimetic LDEV nanodrug delivery system (heparin-cRGD-EVs-doxorubicin, HRED) by modifying heparin-cRGD (HR) on the surface of lemon exosomes and loading them with the chemotherapeutic drug doxorubicin to overcome cancer multidrug resistance (**Figure 5**). This study demonstrated that exosomes can use multiple endocytic routes to enter tumor cells, dissipating intracellular energy and reducing ATP production. This effectively reduces the efflux of drugs by energy-dependent drug-resistant proteins such as P-glycoprotein (P-gp), improving the efficacy of the drug system against drug-resistant cells and significantly inhibiting the proliferation and metastasis of drug-resistant ovarian cancer in nude mice (Xiao et al., 2022).

The poor immunogenic response and the existence of the blood-brain barrier (BBB) present significant challenges for immunochemotherapy of glioma. However, PDVs can effectively bypass the BBB and reprogram the immunosuppressive microenvironment. Li et al. developed four drug-delivery systems based on citrus fruit EVs, with LDEVs showing superior anti-tumor immunity by triggering secondary necrosis. LDEVs significantly promoted dendritic cell maturation, increased cytotoxic T lymphocyte infiltration and reduced the number of regulatory T cells in the glioma microenvironment, thus reversing the immunosuppressive microenvironment (Li et al., 2024).

Kidney stones are a common urinary system disorder, and adequate water intake, along with a combination of fruits and vegetables and citrate, are the main preventive measures. The US Food and Drug Administration has approved potassium citrate for the prevention and treatment of kidney stones. However, long-term use can cause gastrointestinal reactions and increased oxalate excretion, whereas lemon juice does not have these issues. Recently, a research team isolated LDEVs from lemon juice to block the progression of kidney stones. The study found that LDEVs could be transported from the intestine to the kidney, where they were mainly concentrated in renal tubular cells, inhibiting the endoplasmic reticulum stress response in these cells and indirectly interfering with the formation of kidney stones (Zhang et al., 2023).

### 3.8 Broccoli

Broccoli (Brassica oleracea var. italica Plenck), rich in sulforaphane, vitamins, carotenoids, dietary fiber, minerals and flavonoids, is among the most common cruciferous vegetables worldwide. It is nutrient-dense and contains phytochemicals with strong anti-inflammatory, anticancer and antioxidant properties, which can reduce the incidence of cancer, as well as cardiovascular and cerebrovascular diseases (Zandani et al., 2021). Broccoli-derived EVs (BDEVs) have been developed to prevent and treat various diseases (Li et al., 2022).

Constipation is a prevalent gastrointestinal dysfunction that significantly impacts patients’ quality of life and may lead to more serious conditions. Current treatments for constipation often result in drug dependence and side effects. Duan et al. prepared broccoli-derived exosome-like nanoparticles (BDENs), which exhibit high gastrointestinal stability and can alleviate loperamide (LOP)-induced constipation in mice. In this study, oral administration of BDENs (17.5 mg/kg/d) effectively shortened the defecation time, accelerated intestinal propulsion and increased fecal volume in constipated mice (Tianchi et al., 2023).

MiRNAs are small, non-coding RNAs that regulate gene expression but degrade rapidly when unprotected. EVs enhance miRNA stability by protecting them from RNase degradation. Pozo-Acebo et al. prepared BDEVs as a therapeutic vehicle for extracellular RNA drug delivery using ultracentrifugation and SEC. The results demonstrated that BDEVs effectively and stably loaded exogenous miRNAs, which were absorbed by the Caco-2 intestinal cell line and exhibited therapeutic toxicity to these cells (del Pozo-Acebo et al., 2022).

**Figure 6:**
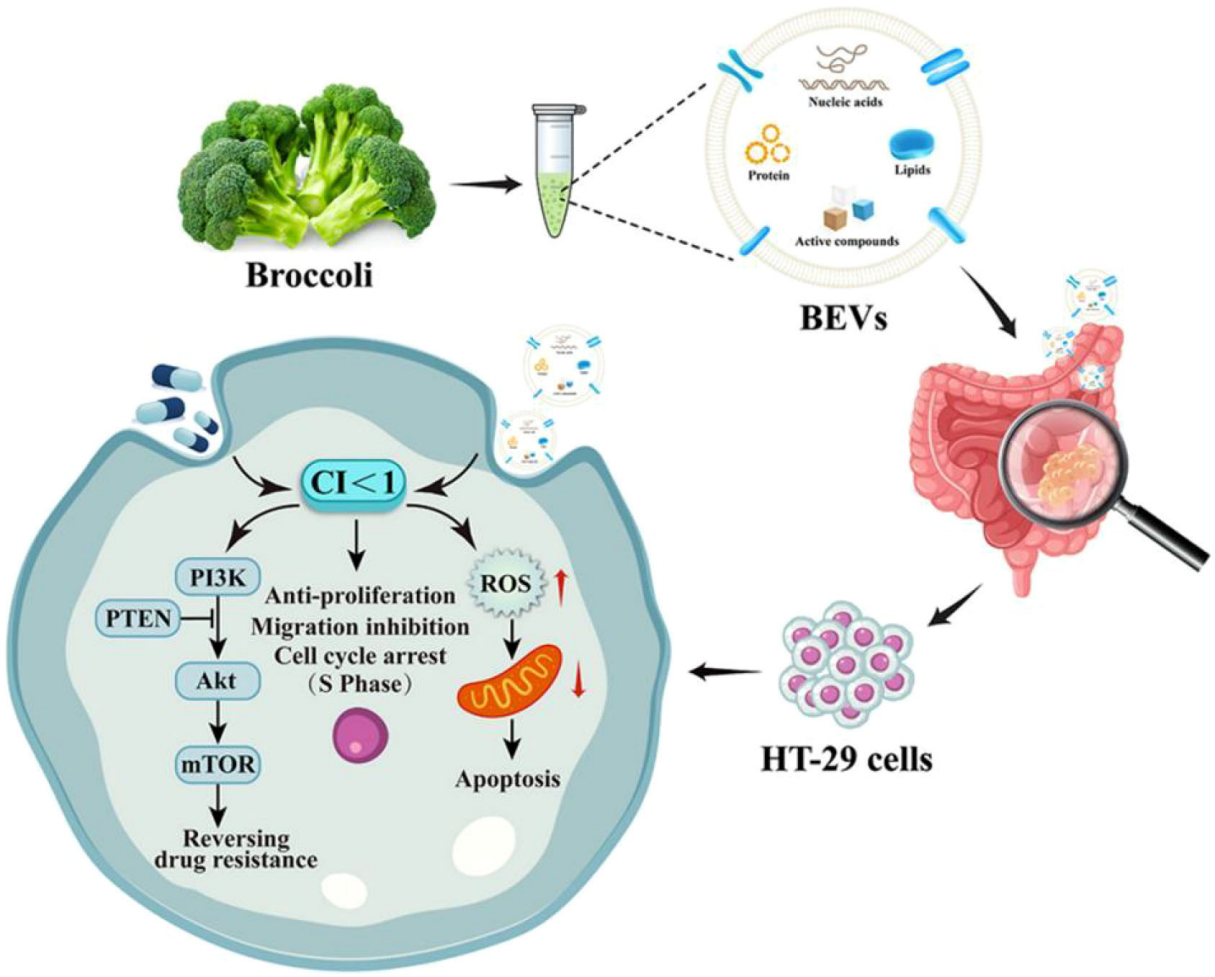
Schematic diagram of broccoli-derived extracellular vesicles and their mechanism in reversing chemoresistance to 5-FU in HT-29 cells. Reprinted with permission from Ref (Cao et al., 2024). Copyright (2024) Elsevier.

FU, a cornerstone of first-line chemotherapy, is effective against colorectal cancer. However, its efficacy is often limited by tumor drug resistance and associated toxicity. Cao et al. extracted BDEVs through differential centrifugation combined with a kit method. The study showed that BDEVs were effectively internalized by colorectal cancer HT-29 cells (**Figure 6**). Co-administration of BDEVs and 5-FU significantly inhibited the proliferation and migration of HT-29 cells, effectively reversing chemoresistance to 5-FU (Cao et al., 2024).

### 3.9 Turmeric

Turmeric, a well-known traditional Chinese medicine with both medicinal and dietary uses, contains numerous compounds with strong antioxidant, anti-inflammatory and anticancer properties. Turmeric-derived nanovesicles (TNVs) have been reported to effectively treat colitis, infections and tumors (Kunihiro et al., 2018).

**Figure 7:**
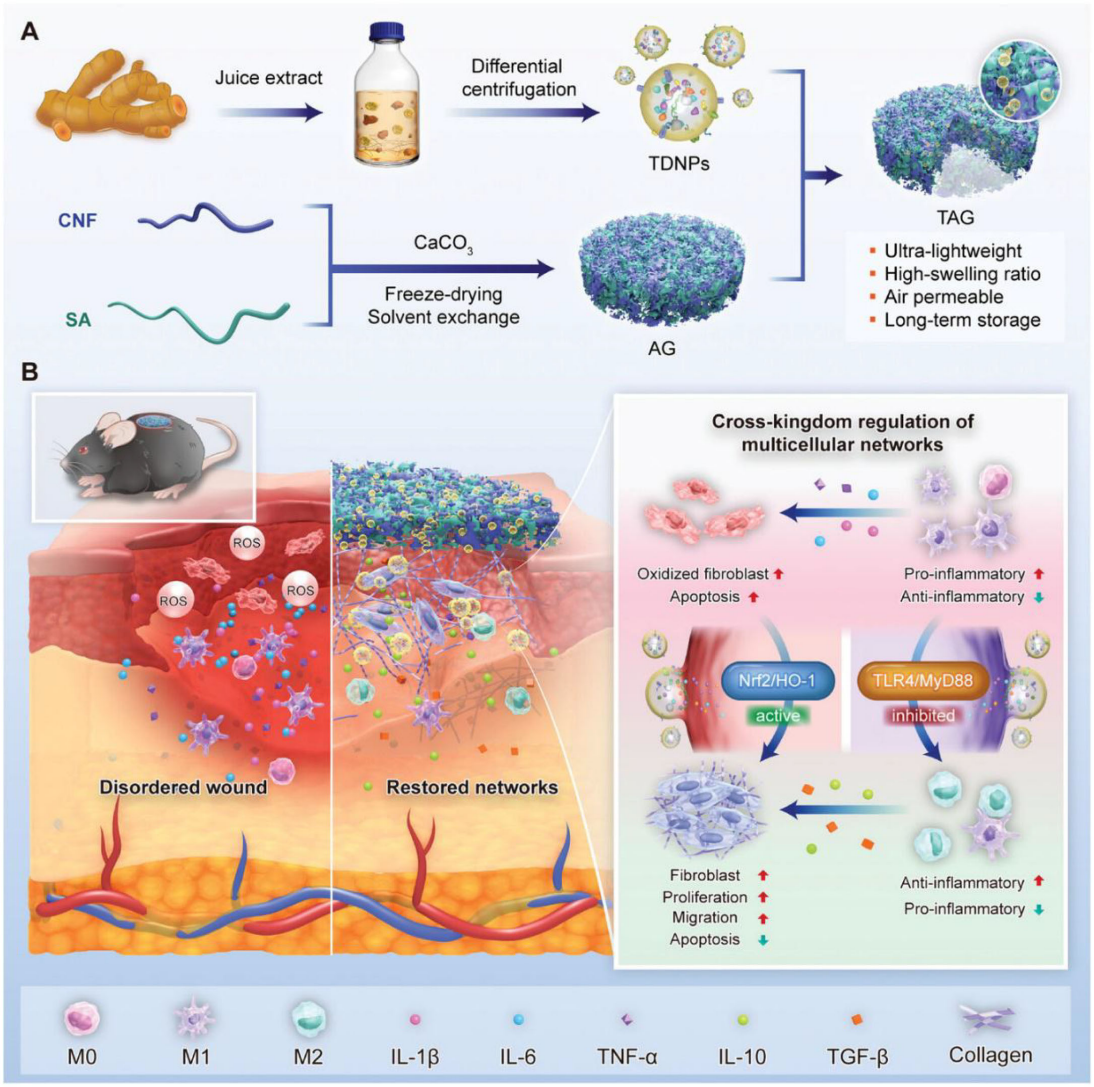
**(A)** Isolation of turmeric-derived nanoparticles (TDNPs) and fabrication of AG and TAG dressings. **(B)** TAG promotes diabetic wound healing by enhancing antioxidant capacity, inhibiting inflammation and restoring multicellular regulatory networks in the wound microenvironment. Reprinted with permission from Ref (Wu et al., 2024). Copyright (2024) Wiley.

**Figure 8:**
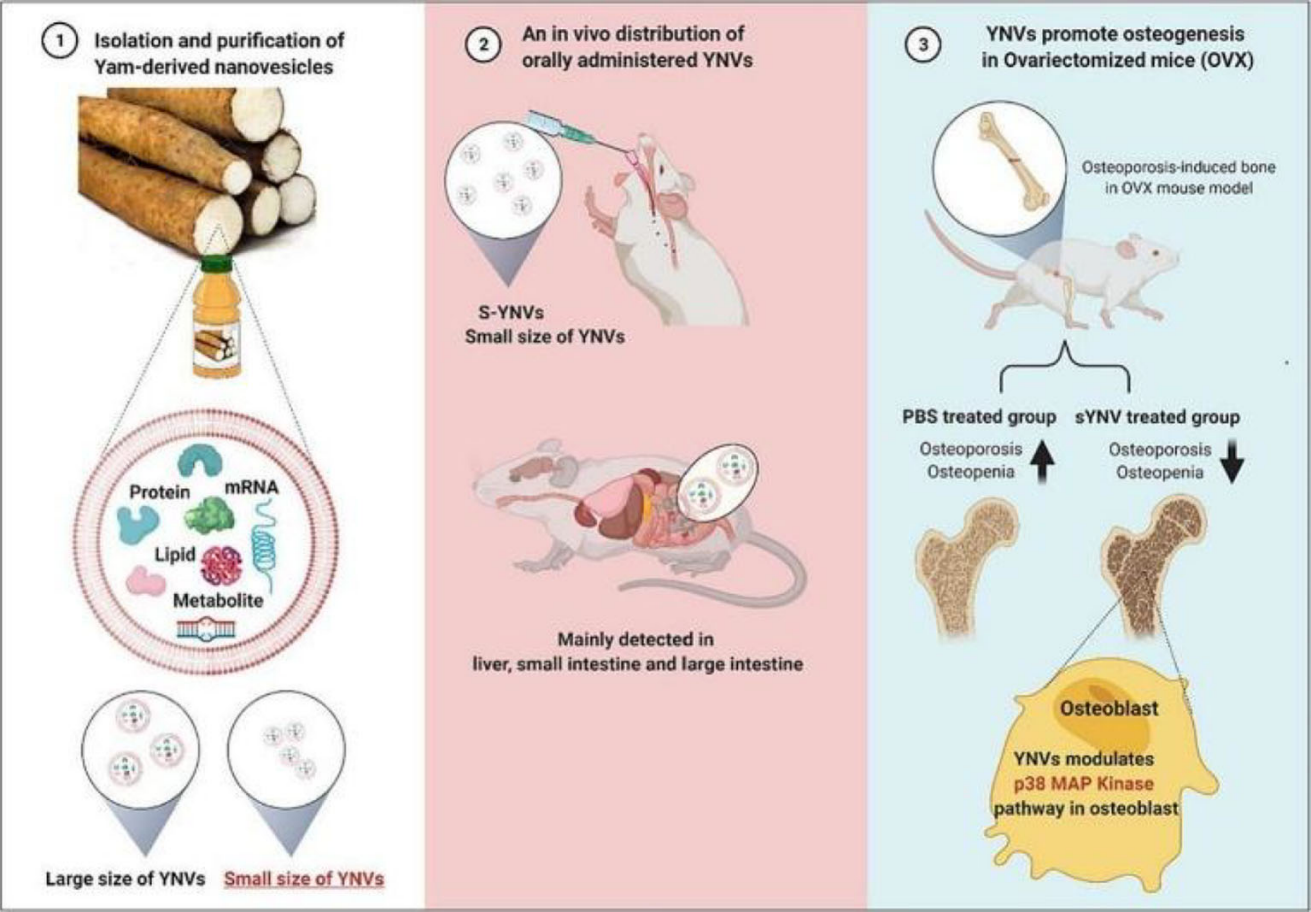
Schematic illustration of the isolation, administration and osteogenic functions of YNVs. Reprinted with permission from Ref (Hwang et al., 2023). Copyright (2023) Elsevier.

Diabetic chronic wounds are among the most serious complications for diabetic patients. Given the substantial social burden and the complex pathophysiological mechanisms involved, there is an urgent need to develop new and effective treatments. Wu et al. developed turmeric-derived nanoparticle (TDNP)-loaded aerogel (TAG) dressings designed to target the wound microenvironment for diabetic wound healing (**Figure 7**). TAG dressings can be customized to fit **Figure 8**. (A) Isolation of turmeric-derived nanoparticles (TDNPs) and fabrication of AG and TAG dressings. (B) TAG promotes diabetic wound healing by enhancing antioxidant capacity, inhibiting inflammation and restoring multicellular regulatory networks in the wound microenvironment (Wu et al., 2024).

Using differential centrifugation, Wei et al. extracted and purified TNVs from turmeric and analyzed their components. They found that TNVs are rich in lipids and various small molecular compounds, which are formed within the rigid bilayer membrane of the vesicles, influencing their secretion and signal transduction (Wei et al., 2023). Additionally, turmeric is known to promote blood circulation and alleviate menstrual-related symptoms. The bioactive molecules in TNVs, enriched in plant exosome particles, suggest that turmeric-derived nanovesicles could serve as novel therapeutic agents for various diseases (Liu et al., 2022).

Ulcerative colitis (UC) is a chronic, idiopathic enteritis that is challenging to treat due to its recurrent nature. Wang et al. designed relevant experiments to explore the pathogenesis and potential treatments for UC. They isolated TNVs from fresh turmeric, purified them using sucrose gradient ultracentrifugation and examined their morphology and structure. A mouse model was then established to study the anti-inflammatory activity and mechanism of TNVs. The findings indicate that TNVs can reduce intestinal inflammatory factor expression, repair the intestinal epithelial barrier and reshape the immune microenvironment, thereby alleviating colitis symptoms and protecting the intestine (Gao et al., 2022).

### 3.10 Other plans

In addition to the numerous studies on PDVs mentioned above, research on plant exosomes has broadened significantly in recent years. More plant-derived exosomes – including those from tomatoes, Flos Sophorae, yams, sesame and Taraxacum mongolicum Hand. (TH) – have been investigated for disease treatment. Spinal cord injury (SCI) is a severe traumatic condition that often leads to complications and multi-organ dysfunction. The sustained release, cellular uptake and long-term retention of therapeutic molecules at the injury site are crucial for continuously improving the microenvironment, necessitating the development of effective drug carriers. Rutin, a compound known to enhance the microenvironment and promote nerve regeneration, was used by Wu et al. to prepare EVs from Flos Sophorae as drug carriers. They then developed a local implantation system for SCI treatment by embedding these vesicles in a polydopamine-modified hydrogel. This approach improved motor function and alleviated dysuria in SCI treatment (Chen et al., 2023).

Lee et al. isolated EVs from tomato fruits through ultracentrifugation and analyzed their physical and morphological characteristics, along with their biocargo profiles. Tomato-derived EVs were found to promote the growth of the probiotic Lactobacillus while inhibiting the growth of opportunistic intestinal pathogens, such as Clostridium difficile and Fusobacterium nucleatum. These findings suggest that tomato-derived EVs have significant potential in treating intestinal microbiota dysbiosis and preventing intestinal bacterial infections (Lee et al., 2023). In another study, tomato-derived EVs were used to accelerate wound healing by enhancing keratinocyte and fibroblast migration (Daniello et al., 2024).

Chinese yam, a highly valued medicinal material from Henan, is widely appreciated for its unique taste and medicinal properties. Yam-derived exosome-like nanovesicles (YNVs), extracted from yam, have been shown to promote osteoblast differentiation and mineralisation in mice with osteoporosis. These YNVs could serve as a safe and effective oral treatment for osteoporosis (**Figure 8**) (Hwang et al., 2023).

Sesame, a significant food crop rich in beneficial compounds such as lipids、polyphenols and polysaccharides, offers various health benefits. Luteolin (Lu), a polyphenol found in plants like pepper and perilla leaves, possesses antioxidative and anti-inflammatory properties. However, its poor water solubility and sensitivity to oxidation and pH limit its stability and bioavailability. Researchers have isolated exosome-like nanocapsules from sesame leaves to encapsulate Lu, significantly improving its solubility and stability, thus offering a novel strategy in the food industry (Jiang et al., 2024).

**Figure 9:**
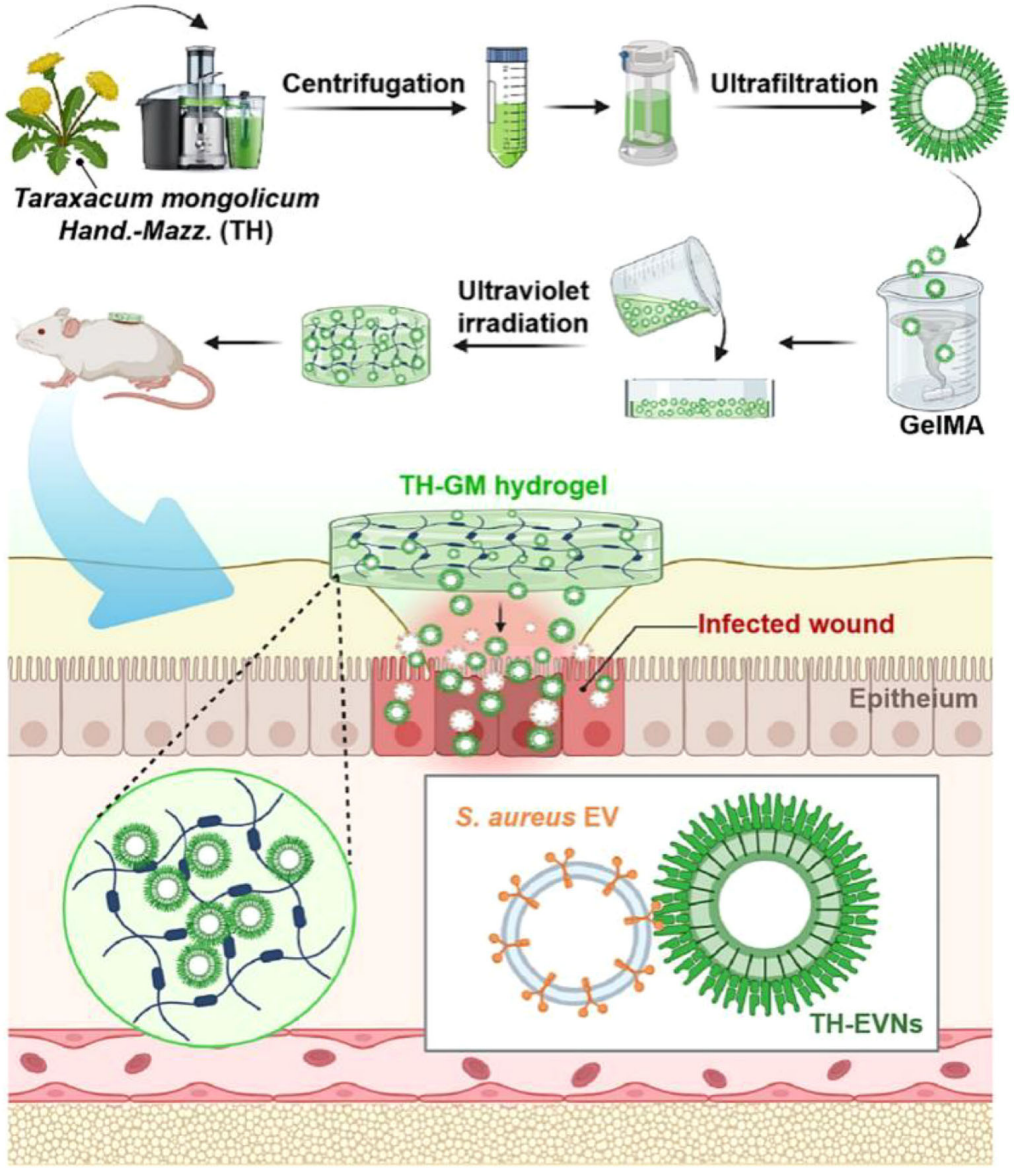
Schematic illustrations of photo-crosslinked hydrogels loaded with dandelion-derived extracellular vesicle-like nanoparticles for treating S. aureus EV-invasive wounds. Reprinted with permission from Ref (Tan et al., 2024). Copyright (2024) Elsevier.

Wound healing impaired by bacterial infection is a major challenge in clinical treatment, and antibiotic abuse remains a significant issue. According to ancient texts, TH is used to treat dermatosis. Applying fresh mashed TH juice to wounds can rapidly alleviate symptoms, promote healing and support recovery from bacterial infections. Exotoxins secreted by Staphylococcus aureus can damage host cells and cause wound infections. Studies have shown that Staphylococcus aureus, which lacks exotoxins, can be efficiently cleared by macrophages. Inspired by this, researchers discovered that extracellular vesicle-like nanoparticles (TH-EVNs) derived from fresh TH could exert antiviral activity by specifically binding to the exotoxins secreted by Staphylococcus aureus, thus protecting host cells and effectively reducing the need for antibiotics.(**Figure 9**) (Tan et al., 2024).

Numerous acute and chronic inflammatory diseases are largely caused by oxidative stress. ROS are crucial for cell signaling at normal levels, but an excess amount can lead to high oxidative stress, damaging proteins, lipids and DNA—ultimately causing cell death. The transcription factor Nrf2 is a key regulator of the antioxidant response, reducing inflammation and injury while protecting cells from oxidative damage by regulating the expression of antioxidant proteins. To develop a material with good biocompatibility, simple synthesis and excellent ROS-scavenging ability, Wan et al. created a novel preparation based on aloe-derived exosome-like nanovesicle-hydrogels (ADENHs) to treat inflammatory diseases associated with ROS. The results showed that both *in vitro* and *in vivo*, ADENHs demonstrated strong antioxidant activity and good biocompatibility. The pathological state of inflammation-related diseases was significantly improved by activating the Nrf2/HO-1 pathway, successfully restoring the balance between oxidation and antioxidant status. This provides a solid scientific foundation for its clinical use (Pan et al., 2024).

## 4 Biomedical applications of PDVs

PDVs are nano-sized vesicles derived from plants with biological activity, promoting host immunity by encapsulating and delivering bioactive molecules to pathogens (Pan et al., 2024). PDVs serve a dual purpose: they contain various bioactive ingredients (e.g. anti-inflammatory, antibacterial and anticancer compounds) that can be used as therapeutic agents, and their nanovesicle structure makes them effective drug carriers (16, Chen and Cai, 2023). PDVs have been widely applied in treating diseases such as inflammation、cancer、oxidative stress、aging and wound healing.

### 4.1 Drug delivery

PDVs, as drug carriers, have a lipid bilayer structure that enables effective encapsulation of both hydrophilic and hydrophobic drugs, enhancing their stability, solubility, bioavailability, immunity and tissue barrier permeability (Shao et al., 2023). Additionally, PDVs can increase drug accumulation in target cells, improving their therapeutic effect (Cao et al., 2023). Compared with synthetic vesicles, PDVs have the advantages of low immunogenicity, fewer side effects, environmental friendliness and excellent biocompatibility, making them a promising and efficient carrier material (Li et al., 2023). As a natural delivery system derived from green plants, the source and natural components of PDVs make them an ideal solution for overcoming the limitations of existing nano-delivery systems (Madhan et al., 2024; Wang et al., 2024).

PDVs, with their good biocompatibility and biodegradability, can be used as drug carriers to load bioactive molecules, enhancing their activity. PDVs can transfer proteins, nucleic acids and other biomolecules into target cells. They can also serve as delivery carriers for RNA molecules, including siRNAs, mRNAs and miRNAs. Four EVs derived from broccoli, pomegranate, apple and orange were extracted using a combination of ultracentrifugation and SEC, serving as nanocarriers for exogenous miRNA delivery. This study demonstrates the potential of PDVs as nanocarriers for miRNA delivery, making them suitable for future RNA-based therapies (de las Hazas et al., 2023). Nucleic acid delivery using PDVs can protect RNA from enzymatic degradation. Orange juice–derived EVs (oEVs) loaded with SARS-CoV-2 mRNA protected their cargo from enzymatic breakdown, remained stable at room temperature for 1 year and triggered a SARS-CoV-2 immune response in mice (Gai et al., 2024). Ganji et al. isolated and characterized nanovesicles (TNVs) from tangerine juice. Metabolomic analysis revealed that TNVs contained flavonoids, organic acids and limonoids. The study showed that TNVs could deliver siRNA to human colorectal cancer cells (Ganji et al., 2024). Yan et al. prepared Brucea javanica–derived exosome-like nanovesicles (BF-Exos) as drug carriers to deliver miRNAs for cancer therapy (Yan et al., 2024). In this study, BF-Exos delivered 10 functional miRNAs to 4T1 cells, significantly inhibiting their growth and metastasis by regulating the PI3K/Akt/mTOR signaling pathway and promoting ROS/caspase-mediated apoptosis. The study showed that BF-Exos can serve as a nano-platform for the delivery of therapeutic miRNAs.

PDVs, as carriers, can be used for drug delivery in addition to nucleic acid delivery. Jiang et al. developed sesame leaf–derived exosome-like nanovesicles (Exo@Lu) as vehicles for Lu delivery. In this study, Exo@Lu demonstrated good stability, enhancing the water solubility and bioaccessibility of Lu (Jiang et al., 2024). Lu et al. prepared celery exosome-like nanovesicles (CELNs) as carriers for the loading of doxorubicin (DOX), obtaining engineered CELNs (CELNs-DOX). CELNs, as drug carriers, showed reduced toxicity and better tolerability in mouse models (Lu et al., 2023).

### 4.2 Anticancer

PDVs, derived from plants, contain various therapeutic and targeted biological activities, including lipids, proteins, nucleic acids and active phytochemicals. These phytochemicals, such as curcumin in turmeric、gingerol in ginger and polyphenols in tea, possess strong anticancer properties, making them valuable for cancer treatment. Several therapeutic systems based on PDVs have been developed for treating breast cancer and liver cancer.

**Figure 10:**
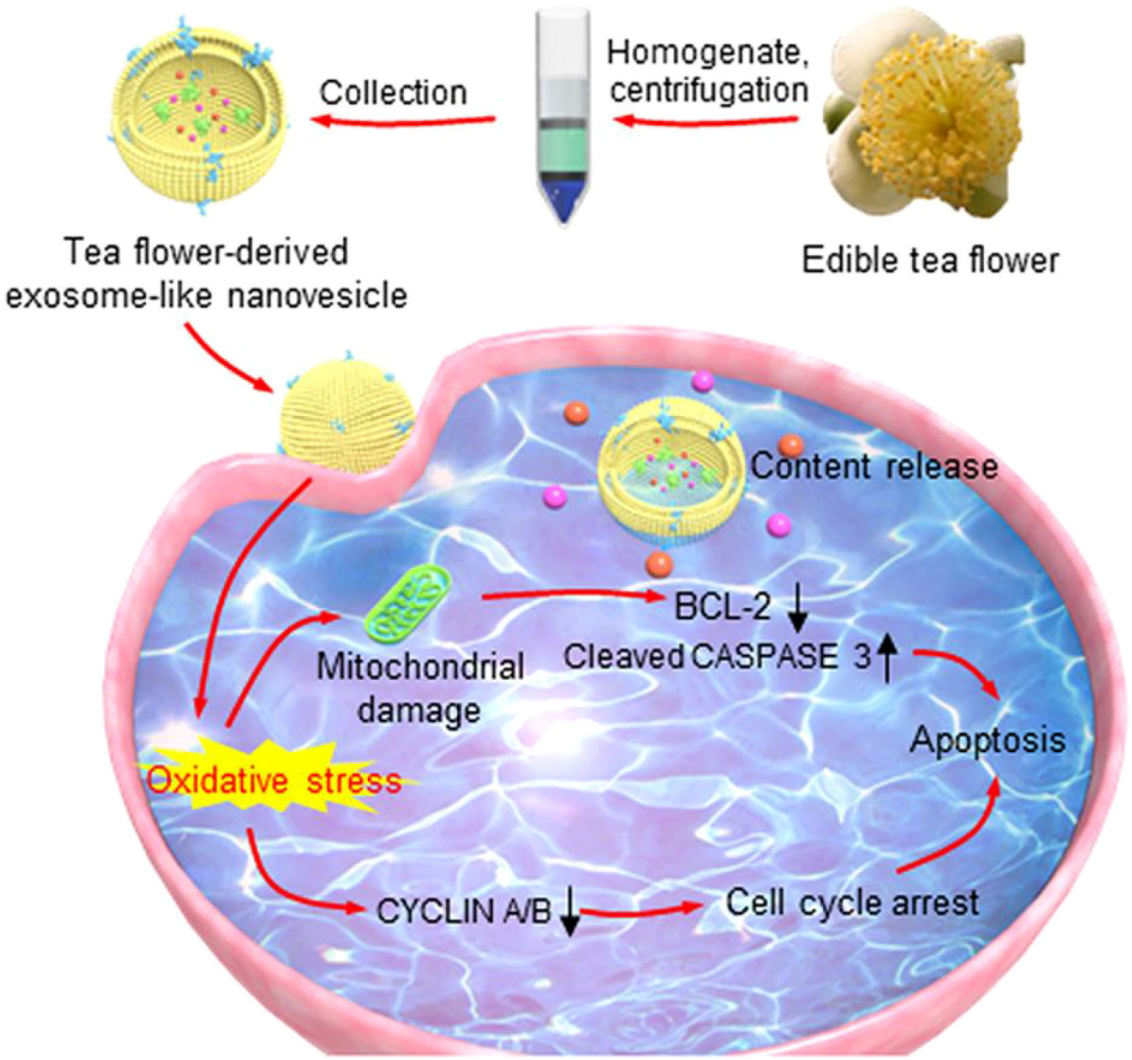
Schematic diagram of edible tea flower–derived vesicles inhibiting breast cancer metastasis. Reprinted with permission from Ref (Chen et al., 2022) Copyright (2022) Elsevier.

**Figure 11:**
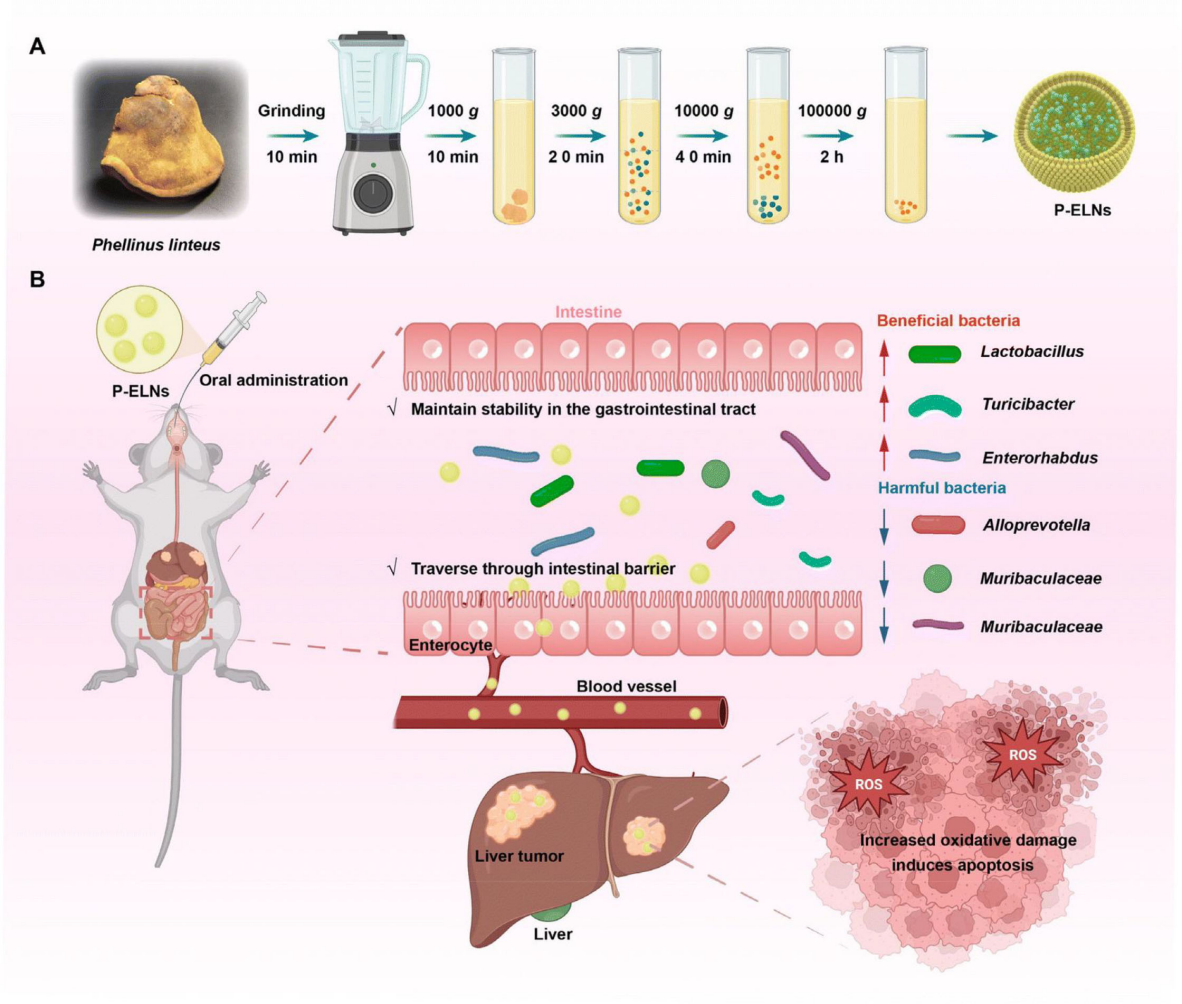
Schematic diagram of P-ELN preparation and anti-liver cancer activity. **(A)** Method for isolating and purifying P-ELNs from Phellinus linteus. **(B)** P-ELNs promote apoptosis, increase the abundance of beneficial bacteria and decrease the quantity of harmful bacteria. Reprinted with permission from Ref (Zu et al., 2024). Copyright (2024) Royal Society of Chemistry.

Chen et al. developed exosome-like nanovesicles derived from edible tea flowers (TFENs) to suppress metastatic breast cancer through ROS generation and microbiota modulation(**Figure 10**). They found that TFENs contained various polyphenols, flavonoids, functional proteins and lipids. TFENs induced mitochondrial damage, cell cycle arrest, apoptosis and microbiota modulation, further inhibiting the growth of breast tumors and their lung metastasis (Chen et al., 2022). Xiao et al. extracted and purified natural exosome-like nanoparticles from Phellinus linteus (P-ELNs) to suppress metastatic hepatocellular carcinoma through ROS generation and microbiota rebalancing (**Figure 11**). Oral administration of P-ELNs improved therapeutic outcomes in an animal model of metastatic hepatocellular carcinoma by amplifying ROS and rebalancing the gut microbiome. These findings suggest that P-ELNs can serve as a promising oral therapeutic platform for liver cancer treatment (Zu et al., 2024). Similarly, Xiao et al. extracted and purified natural exosome-like lipid nanoparticles from black mulberry (Morus nigra L.) (MLNPs) leaves for targeted oral treatment of hepatocellular carcinoma. This study demonstrated that MLNPs serve as a natural, safe and robust nanomedicine platform for the oral treatment of hepatocellular carcinoma (Gao et al., 2024). Bitter melon, a medicinal and edible plant with anti-tumor effects, was used by Liu et al. to isolate a bitter melon–derived vesicle extract (BMVE) from bitter melon juice through ultracentrifugation for treating breast cancer (Feng et al., 2023).

### 4.3 Anti-inflammatory

Inflammation is typically treated with anti-inflammatory drugs, but prolonged use of these drugs can increase the risk of adverse side effects. PDVs are cell-secreted vesicles with strong anti-inflammatory properties and have been used in many studies to treat inflammatory diseases. They may exert their anti-inflammatory effects through various mechanisms, such as modulating the immune system, inhibiting inflammatory factors and reducing oxidative stress. Many PDVs have been developed to treat various inflammatory diseases, including colitis (Cai et al., 2022; Zhang et al., 2024).

IBD is a chronic, recurrent intestinal condition with a complex etiology. Currently, treatment aims to alleviate symptoms but offers no effective cure. Recent studies have shown that PDVs can potentially deliver drugs targeting the intestine and show great promise for treating intestinal diseases (Singh et al., 2024). Wang et al. developed ginseng-derived nanoparticles for treating IBD. These GDNPs alleviate IBD via the TLR4/MAPK and p62/Nrf2/Keap1 pathways (Yang et al., 2024). IBD may also be associated with lung inflammation and tissue damage. Pueraria lobata (P. lobata) plays an important role in controlling cytokines. P. lobata–derived exosome-like nanovesicles (PLDENs) were constructed to treat colitis-associated lung inflammation. The study investigated the effects of PLDENs on colitis and their role in the lung inflammatory response (Lu et al., 2024). Liu et al. found that 82% of cabbage PDVs were destroyed under conditions simulating the upper digestive tract, so they developed an oral delivery system by loading stabilized EudragitS100-coated cabbage PDVs into capsules (Cap-cPDVs). Cap-cPDVs showed robust therapeutic effects in a dextran sulfate sodium-induced colitis mouse model (Liu et al., 2023). Emmanuela et al. prepared exosome-like nanoparticles derived from black nightshade berries (Solanum nigrum L.) (PDENs) as an anti-inflammatory agent. The results demonstrated that the PDENs could exert anti-inflammatory activity by suppressing IL-6 production in LPS-stimulated RAW264.7 cells (Emmanuela et al., 2024).

### 4.4 Antioxidant

PDVs can treat various skin diseases because they contain a range of bioactive molecules from plants, including antioxidants. Different plants contain different types of antioxidants, mainly depending on their types and sources. Among these, vitamin C, carotenoids and phenolic compounds are the primary antioxidant phytochemicals from plants (Kim et al., 2023a). For a long time, these antioxidants have been extracted from plants using various methods and have been used to treat diseases caused by oxidative stress, including carcinogenesis and skin aging (Cui et al., 2020).

Iris species is a traditional Chinese medicine, and its extract exhibits anti-inflammatory, antioxidant and antibacterial activities. Researchers analyzed the components of Iris germanica L. and identified several antioxidant compounds, including flavonoids and triterpenoids. Additionally, many phenols, ceramides and benzoquinone derivatives were found. It is, therefore, reasonable to consider Iris exosomes as a potential source of anti-aging drugs targeting intracellular ROS (Amin et al., 2021). Kim et al. studied the protective effect of Iris exosomes from the rhizome of Iris germanica on aging dysfunction in human epidermal keratinocytes (NHEKs) induced by oxidative stress. In this study, hydrogen peroxide (H_2_O_2_) was used to induce oxidative stress in keratinocytes. The use of Iris exosomes significantly reduced the ROS levels induced by H_2_O_2_ in NHEKs and degraded the H_2_O_2_-induced sa-β-gal-positive NHEKs. These results suggest that Iris exosomes may act as antioxidants and anti-aging agents, preventing dysfunction in NHEK cells induced by oxidative stress (Kim et al., 2023).

UC is a chronic, non-specific intestinal disease characterized by diarrhea, abdominal pain, bloody stools, intestinal inflammation and epithelial destruction, which can lead to severe disability. Currently, the etiology and mechanisms of UC remain unclear, and treatment strategies only aim to alleviate symptoms and reduce patient suffering. Bitter gourd, known for its bitter taste, is highly valued for its nutritional and medicinal properties. Wang et al. demonstrated that EVs derived from Momordica charantia (MCEVs) exhibit strong antioxidant properties and can help restore gut health in UC by regulating oxidative stress and inflammatory factors. This study suggests that MCEVs, as drug carriers, offer promising new insights for UC treatment (Wang et al., 2023).

### 4.5 Wound healing

As the largest organ in the human body, the skin plays a crucial role in regulating body temperature and water balance and protecting internal organs from the external environment. Skin injury can lead to severe physiological imbalances, bacterial infections, inflammation and disability. Therefore, wound healing is vital for human health. Wound healing consists of two main stages: the first is coagulation, where platelets form clots to prevent foreign substances from entering, and the second is the inflammatory response, which removes invading bacteria. During the entire healing process, low levels of ROS promote wound healing more effectively than other conditions, aiding recovery (Wang et al., 2023). PDVs not only have anti-inflammatory effects but also promote cell proliferation and angiogenesis, making them a valuable biomaterial for wound healing. For example, tomato-derived nanovesicles (TDNVs) accelerate wound healing by inducing the migration of keratinocytes and fibroblasts. TDNVs also exhibit antiviral activity by specifically binding to the exotoxins secreted by Staphylococcus aureus, thus reducing their levels (Mammadova et al., 2023).

Platycodon grandiflorum, a perennial plant in the Campanulaceae family, contains bioactive compounds, including platycodin, flavonoids and phenolic acids, which demonstrate anti-inflammatory and antioxidant effects. Kim et al. isolated extracellular vesicles from balloonflower root (BFR-EVs) using PEG precipitation and ultracentrifugation. Their study found that BFR-EVs inhibited the expression of pro-inflammatory cytokine genes in lipopolysaccharide-stimulated cells and alleviated inflammation. Additionally, BFR-EVs promoted the migration of human dermal fibroblasts and showed proliferative effects in the water-soluble tetrazolium salt-8 assay. Compared with other drugs, BFR-EVs demonstrated lower toxicity, making them a promising natural treatment for chronic skin wounds (Kim et al., 2023b).

Aloe barbadensis possesses antioxidant, anti-inflammatory, antibacterial and anti-aging properties and is commonly used to treat burns. Kim et al. isolated Aloe barbadensis–derived extracellular vesicles (AS-EVs) by precipitating PEG, then treated RAW264.7 macrophages stimulated with LPS using AS-EVs. They observed a significant decrease in the expression of pro-inflammatory genes, demonstrating a strong anti-inflammatory effect. In addition, AS-EVs were tested in two assays: the water-soluble tetrazolium salt-8 and transwell migration assays. The results showed that AS-EVs stimulated blood vessel formation and enhanced the proliferation of human dermal fibroblasts, which supports chronic wound healing (Kim and Park, 2022).

## 5 Summary and future prospects

In recent years, research on plant - derived vesicles (PDVs) has witnessed a remarkable upsurge, propelled by their extraordinary attributes, including high safety, targeted delivery proficiency, natural intercellular communication superiority, and extensive *in vivo* bioactivity (Cao et al., 2023). Despite the promising prospects of PDVs, a number of crucial challenges hamper their clinical translation.

Firstly, the lack of standardized methodologies for PDV isolation and characterization results in substantial disparities among studies, rendering comparisons arduous. Secondly, the underlying mechanisms of PDV biosynthesis in plants, their cellular uptake in mammals, and subsequent intracellular trafficking are still inadequately comprehended. Although certain studies propose endocytic pathways, the specific receptors and signaling cascades involved demand further clarification. Thirdly, scaling up PDV production while maintaining consistency in size, composition, and functionality poses considerable technical hurdles. Current isolation methods are predominantly devised for laboratory - scale production and may not be economically feasible for industrial applications. Finally, regulatory frameworks for plant - derived nanovesicles as therapeutic agents are almost non - existent, generating uncertainty for clinical development. Addressing these challenges necessitates collaborative endeavors among plant biologists, nanotechnologists, and clinical researchers to establish standardized protocols, enhance mechanistic understanding, develop scalable production technologies, and interact with regulatory agencies to define approval pathways.

Furthermore, while seasonal and regional factors may influence plant bioactive components, the primary determinant of PDV functionality appears to be the plant species itself. Therefore, future efforts should prioritize the establishment of standardized evaluation protocols for PDVs to ensure consistency and efficacy in developing targeted plant-based therapies.A standardized evaluation system for PDVs should include three key parts: (1) standardizing plant sources by selecting varieties with stable yield and bioactivity and optimizing cultivation conditions; (2) unifying preparation parameters (e.g., differential centrifugation, buffer composition, purification) to ensure reproducibility; (3) adopting appropriate characterization methods for quality control (particle size distribution, zeta potential, bioactivity) to guarantee therapy safety and efficacy.

The clinical translation of plant-derived vesicles (PDVs) encounters several obstacles beyond technical challenges. Safety considerations encompass potential immunogenicity, particularly during repeated administration. Although current evidence indicates that PDVs exhibit lower immunogenicity compared to mammalian extracellular vesicles (EVs). Batch-to-batch variability resulting from plant growth conditions, harvesting time, and processing methods presents substantial challenges for quality control. Regulatory agencies have not yet formulated clear guidelines for plant-derived nanotherapeutics, leading to uncertainty in product development. Additionally, intellectual property issues related to natural products may complicate commercial development. Future research should tackle these translational challenges through systematic toxicology studies, standardization of production processes, and early engagement with regulatory bodies during the development process.
